# Dioxin-like Activity in Pregnant Women and Indices of Fetal Growth: The ACCEPT Birth Cohort

**DOI:** 10.3390/toxics10010026

**Published:** 2022-01-08

**Authors:** Manhai Long, Maria Wielsøe, Eva Cecilie Bonefeld-Jørgensen

**Affiliations:** 1 Centre for Arctic Health and Molecular Epidemiology, Department of Public Health, Aarhus University, 8000 Aarhus C, Denmark; mwielsoe@ph.au.dk (M.W.); ebj@ph.au.dk (E.C.B.-J.); 2 Institute of Nursing and Health Science, Greenland Center for Health Research, University of Greenland, Nuuk 3905, Greenland

**Keywords:** persistent organic pollutants, dioxin-like activity, aryl hydrocarbon receptor, Greenlandic Inuit, pregnant women, fetal growth indices

## Abstract

Exposure to lipophilic persistent organic pollutants (lipPOPs) elicits a number of species- and tissue-specific toxic responses, many of which involve the aryl hydrocarbon receptor (AhR). This study aims to measure the combined serum dioxin-like activity of lipPOPs in Greenlandic Inuit pregnant women and the associations with fetal growth indices. The combined dioxin-like activity of serum lipPOPs extracts was determined using the AhR reporter gene bioassay and expressed as pico-gram (pg) TCDD equivalent (TEQ) per gram serum lipid [AhR-TEQ (pg/g lipid)]. Significant AhR-TEQ was found in >87% of serum samples with the median level of 86.2 pg TEQ/g lipid. The AhR-TEQ level positively correlated with the marine food intake biomarker n-3/n-6 polyunsaturated fatty acids ratio, while negatively correlated with body mass index and parity. Women giving birth to infants with low birth weight (<2500 g) and length (<50 cm) had higher AhR-TEQ level compared to those with normal weight and length infants. For previous smokers, we found significant inverse associations between maternal AhR-TEQ level and fetal growth indices. In conclusion, exposure of Greenlandic Inuit pregnant women to dioxin-like compounds through traditional marine food can adversely influence the fetal growth via induced AhR activity. Smoking might have modifying effects.

## 1. Introduction

The lipophilic persistent organic pollutants (lipPOPs) include dioxins [polychlorinated dibenzo-p-dioxins/furans (PCDDs/PCDFs)], polychlorinated biphenyls (PCBs), organochlorine pesticides (OCPs) and polybrominated diphenyl ethers (PBDEs). Because of resistance to degradation, lipPOPs bio-accumulate and magnify in animals, e.g., marine mammals and humans through the food chain [1,2,3]. Due to their lipophilic characteristics and long-range atmospheric and oceanic transport [4], the Greenlandic Inuit is to a greater extent exposed to lipPOPs through high intake of traditional foods, i.e., marine food such as whale, seal, walrus and polar bear [5,6,7]. The ratio between n-3 polyunsaturated fatty acids (PUFA) and n-6 PUFA (n-3/n-6 PUFA ratio) is a strong indicator of marine food intake [8] and used as an indicator of consumption of Greenlandic traditional food versus imported food.

Exposure to dioxins, e.g., 2,3,7,8-tetrachlorodibenzo-p-dioxin (TCDD), and dioxin-like compounds (DLCs) such as non-ortho and mono-ortho PCBs may cause a series of negative effects, both observed in animal experiments and in human epidemiological studies including carcinogenicity [9,10], immune toxicity and adverse effects on reproduction and neurobehavioral [11]. The toxicity of dioxins and DLCs is mediated by binding to the aryl hydrocarbon receptor (AhR), an intracellular ligand-dependent transcriptional factor expressed in most tissues of mammals [12]. The activation of AhR causes induction of gene transcription, for instance, encoding for metabolizing enzymes [13]. A variety of naturally occurring dietary AhR ligands (e.g., indoles, tetrapyrroles, arachidonic acid metabolites and flavonoids) have been identified [14]. Apart from its role as a mediator of the toxic response to xenobiotics, AhR plays a key endogenous regulatory role in normal physiology and development [14]. Previous studies showed a two-way cross talk between the intracellular signaling pathways involving the estrogen- (ER) and androgen- (AR) receptor, as well as the AhR [15]. Interference of persistent organic pollutants (POPs) or their metabolites with hormone receptors has also been observed [16,17] and thus can affect reproductive and endocrine functions [18,19].

Because dioxins and DLCs exist as complex mixtures of various congeners throughout the environment, the TCDD toxic equivalent (TEQ) has been used to simplify the risk assessment and regulatory control [3]. Calculation of the classical TEQs is relying on the concentrations of the individual PCDDs/PCDFs/DL-PCBs and their respective toxic equivalency factors (TEFs). However, previous studies emphasizes that assessment of the toxicological potential of a chemical mixture is much more complex than can be deduced by a given calculated TEQ value using TEFs [19,20]. There are several drawbacks using the TEF concept for risk assessment of POP mixtures, e.g., (i) expensive and time consuming gas chromatography-mass spectrometry determinations; (ii) low concentrations of individual congeners which are below the limit of detection (LOD) of instruments; (iii) the presence of compounds which cannot be routinely measured or unknown compounds having AhR affinity; (iv) no TEF values for several POPs; and (v) not considering the possible antagonistic or synergistic interactions between POPs [21,22,23]. Therefore, to measure the combined dioxins and DLCs activity, we used the in vitro AhR trans activity reporter-gene bioassay. This bioassay is proven to be a quick and sensitive assay to detect the AhR mediated potential of pure chemicals [21,22,23,24], extracts of environmental and biological specimen, and thus the integrated TEQ value (AhR-TEQ) of complex mixtures found in pore water, sediment, milk from bovine and human, human serum and follicular fluid [25]. To remove undesired AhR active substances such as polycyclic aromatic hydrocarbons (PAHs) and naturally occurring dietary AhR ligands, the bioassay can be coupled with an extensive clean-up procedure [25], insuring that mainly the stable lipophilic compounds such as POPs are measured.

Studies showed that the seafood intake of pregnant women in Denmark and USA correlated to their blood levels of lipPOPs including dioxins and DLCs [26,27]. During pregnancy, dioxins and DLCs can be transferred from the mother via the placenta to the fetus and may lead to a variety of toxic effects [28]. Animal studies showed a link between high prenatal exposure to TCDD and commercial PCB mixtures containing dioxin-like PCBs (DL-PCBs) and reduced birth size in the offspring [29,30]. A few epidemiological studies have investigated prenatal or perinatal exposure to dioxins and fetal growth and the most commonly measured parameters of fetal growth are birth weight (BW), birth length (BL), head circumference (HC) and gestational age at birth (GA) [31,32,33,34,35,36,37]. The majority of epidemiology studies evaluate the effect of exposure to individual dioxins and DL-PCBs congeners during pregnancy in relation to fetal growth [32,36,38,39,40,41]. Few studies assessed the association of fetal growth and the combined effect on AhR of dioxin and DLC mixtures existing in the mother’s blood by the measuring AhR-activity, and inconsistent results were shown [42,43].

The prospective and geographical mother-child cohort ACCEPT (Adapting to Climate Change, Environmental Pollution and Dietary Transition) was established in Greenland in 2010–2015 to evaluate the lifestyle, diet and exposure levels of POPs during pregnancy, and assess their possible association with the fetal development and infant/child health [44,45,46]. The present study aims to measure the combined lipPOP induced dioxin-like activity in maternal serum of the ACCEPT pregnant women using an AhR transactivity reporter gene bioassay and evaluate the associations between dioxin-like activity and fetal growth indices including BW, BL, HC, GA and ponderal index (PI). PI is a measure of intrauterine growth for infants.

## 2. Materials and Methods

### 2.1. Study Population

Recruitment of the pregnant women for the mother-child cohort occurred between August 2010 and August 2011 and between June 2013 and September 2015. The enrollment depended on coast visits carried out by a clinical doctor (2010–2011) and time available for the midwives (2013–2015). Thus, we used a convenient sampling method to cover the very wide different geographical parts of Greenland. Therefore, it was not possible to recruit and invite 1879 pregnant women. The 614 pregnant women were recruited during 2010–2015 [44,45,46]. Inclusion criteria was that participants had to be ≥18 years of age, lived more than 50% of their lives in Greenland and to be Inuit defined as having at least one parent born in Greenland and/or have Greenlandic as their native language.

**Figure 1:**
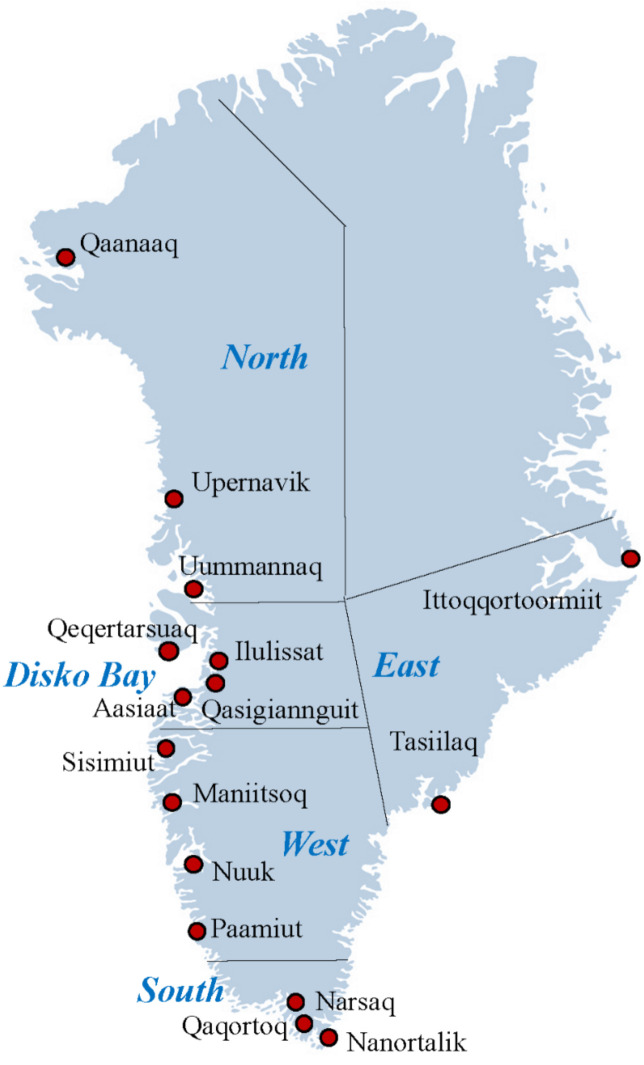
Map of Greenland with the data collection sites. Map source: Modified from colourbox.com.

**Figure 2:**
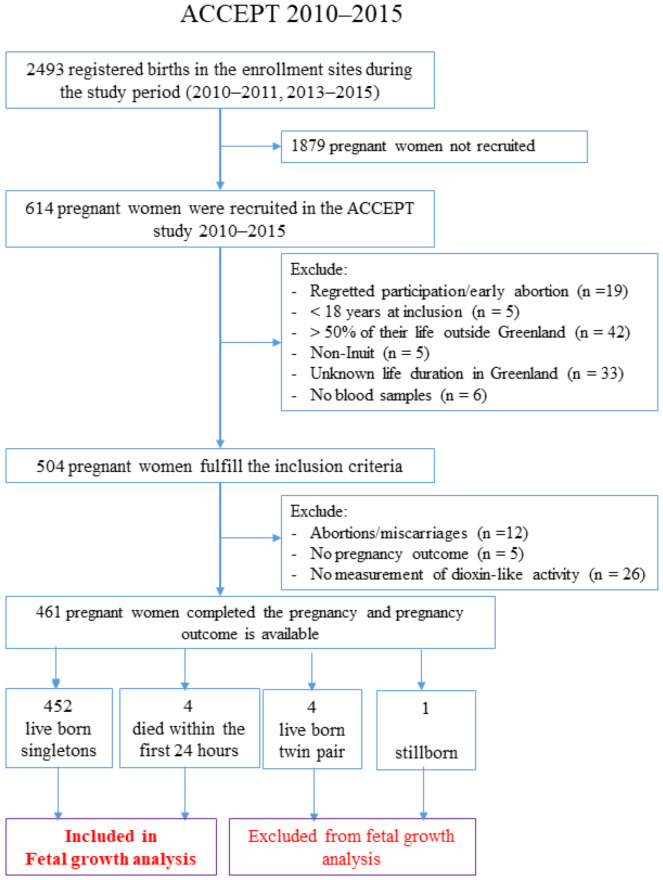
Flowchart for inclusion of study participants.

Clinical doctors and midwives recruited participants in 16 towns from all regions in Greenland: North (Qaanaaq, Upernavik, Uummannaq), Disko Bay (Qeqertarsuaq, Ilulissat, Aasiaat, Qasigiannguit), West (Sisimiut, Maniitsoq, Nuuk, Paamiut), South (Narsaq, Qaqortoq, Nanortalik), East (Tasiilaq, Ittoqqortoormiit) (Figure 1). In total 504 participants meet the inclusion criteria (Figure 2) by excluding 110 participants: 19 regretted participation or had an early abortion/miscarriage; 5 was below 18 years of age; 42 had lived for more than 50% of their lives outside of Greenland; 5 were non-Inuit, 33 missed information on life duration in Greenland and 6 had no blood samples. During pregnancy, 12 women had abortions or miscarriages and for five women there were no birth outcome data. In addition, no dioxin-like activity data were available for 26 women due to not enough sample volume. Furthermore, we excluded four twin pairs and one stillborn. This left 456 mother-child pairs available for analysis of maternal dioxin-like activity and birth outcomes (Figure 2).

All participants gave an informed consent prior to data collection. The women filled in lifestyle questionnaires offered in both Greenlandic and Danish language. The questionnaire provided information about the place where the women were born, place where they grew up and had lived. The women belonged to the region where they had lived longest. Data from medical records and lifestyle questionnaires included pre-pregnancy body mass index (BMI), smoking history, alcohol intake, education level and reproductive factors [44,46]. At inclusion, venous blood samples were taken from the pregnant women. Mean gestational week of sampling in 2010–2011 was week 26.2 (range 7–40 weeks) and in 2013–2015 all samples were collected before the end of week 13 [44,46]. The samples were stored at −80 °C until analysis.

The measurements of the newborns were carried out by midwives at birth, and data on fetal growth obtained from the Chief Medical Office of Greenland.

The Ethical Committee for Scientific Investigations in Greenland approved the study conducted in accordance with the Helsinki Declaration.

### 2.2. Plasma Fatty Acids Easurement

The n-3 and n-6 PUFA measurements were performed at Lipid Analytical Laboratories, Guelph, ON, Canada. Details on analytical methods and measurement methods was published previously [45].

### 2.3. Plasma Cotinine Levels Measurement

Calbiotech Cotinine Direct ELISA Kit (Calbiotech Inc., El Cajonm, CA, USA) was used to measure the cotinine concentrations at Centre of Arctic Health and Molecular Epidemiology, Aarhus University, Denmark. Levels were given in ng/mL and the LOD was 1.0 ng/mL. If the values were below the LOD, 0.5 ng/mL was used for the statistical analysis.

### 2.4. Measurement of Lipophilic POPs and Metals

The materials and sample preparation followed the guide from the analyzing laboratory, Centre de Toxicologie, Institute National de Santé Publique du Québec, Canada, a contract laboratory certified by the Canadian Association for Environmental Analytical Laboratories [47]. This contract laboratory is an international quality-control laboratory that send test samples to an array of international laboratories for quality-control. The serum samples (2 mL) for lipPOPs measurements from the pregnant women were pipetted in the supelco glass vials cleaned by ethanol and hexane and stored in the freezer and then further sent to the analyzing laboratory with dry ice. The lipPOPs were analyzed on an Agilent 6890 or 7890 gas chromatograph (GC) equipped with an Agilent 7683 series or 7693 automatic injector and an Agilent 5973 Network or 5975C mass spectrometer (MS) and serum total lipids were measured using a standard enzymatic procedure at Centre de Toxicologie, Institute National de Santé Publique du Québec, Canada [48,49]. The analyses included 11 OCPs (Aldrin, Alphachlordane, cis-Nonachlor, γ-Chlordane, Hexachlorobenzene (HCB), Mirex, Oxychlordane, Dichlorodiphenyldichloroethylene (p,p’-DDE), Dichlorodiphenyltrichloroethane (p,p’-DDT), β-Hexachlorocyclohexane (β-HCH) and trans-Nonachlor); 14 PCBs (28, 52, 99, 101, 105, 118, 128, 138, 153, 156, 170, 180, 183 and 187), polybrominated biphenyl 153 (PBB 153), and 9 PBDEs (15, 17, 25, 28, 33, 47, 99, 100 and 153).

The whole blood levels of metals including mercury (Hg), selenium (Se) and plasma Se were measured inductively coupled plasma mass spectrometry at the accredited element laboratory, Institute for Bioscience, Arctic Research Centre, Aarhus University, Denmark [50].

### 2.5. Serum Sample Extractions for Determination of AhR Mediated Dioxin-like Activity

Aliquots of serum (2 mL) were mixed with 2 mL 90% formic acid and degassed. The samples were then extracted using solid phase extraction (SPE) by loading onto a conditioned Discovery DSC C18 SPE column (6 mL, 500 mg, Sigma-Aldrich) and passed through. Then the SPE columns were washed with 2 × 3 mL 5% methanol and dried for 2 h using vacuum. By adding 2 × 3 mL hexane, the extracts components were eluted from the SPE column, and collected in clean brown tubes to avoid light. Subsequently, the SPE extracts were loaded on to a conditioned Supelco multi-layer silica column and florisil column (Sigma-Aldrich, Darmstadt, Germany. The columns were eluted with 175 mL n-hexane followed with 25 mL n-hexane: dichloromethane (98:2, *v*/*v*) and 50 mL dichloromethane. The final extracts were evaporated in a rotary evaporator to about 2 mL, stored in clean brown vials at room temperature in the dark under ventilation and the day before the AhR transactivation bioassay the extract was further evaporated to near dryness using N_2_. The final extract was resuspended in solution of dimethyl sulfoxide and water [DMSO: H_2_O (1:1)] and stored overnight for the AhR transactivation bioassay [51].

For each set of seven serum samples, a procedure blank (2 mL of 3 × distilled water) and a control serum (pooled human serum samples from the municipal hospital blood bank, Aarhus, Denmark) were in parallel extracted and clean-up in the same way as the test serum samples.

### 2.6. Measurement of Dioxin-like Activity Using AhR Transactivation Assay

Detailed procedure of AhR transactivitation assay was described elsewhere [52,53]. Briefly, we used the stable transfected mouse hepatoma cell line Hepa1.12cR carrying the AhR-luciferase reporter gene. In each assay, on each plate in parallel we tested the control samples: the solvent control (SC) (sample solvent alone used for dissolving the serum extract pellet) as negative control and the half-maximum effect concentration of TCDD (EC_50_-TCDD) as positive control.

The Hepa1.12cR cells were exposed in parallel to the control samples (SC and EC_50_-TCDD), extracts of test serum samples, procedure blank (3 × distilled water) and control serum (pooled biobank serum). After 4 hours exposure, cells were harvested, and luciferase activity was measured and expressed as relative light unit (RLU) per count protein (RLU/count protein). In each independent assays, the mean luciferase activity (RLU/count protein) of SC was set to 1. We determined the dioxin-like activity of EC_50_-TCDD, extracts of procedure blank and control serum, and the test serum samples by the ratio of their luciferase activities above luciferase activity of SC. No contamination in the extraction procedure was observed, as the dioxin-like activity of procedure blank was lower than the control serum.

In each experiment, we analyzed in parallel a 9-point of TCDD titration dose-response curve at concentrations ranging from 2 × 10^−12^ to 5 × 10^−9^ M. The EC_50_-TCDD was calculated to be 1.8 × 10^−10^ M by fitting the TCDD dose-response data into a three-parameter sigmoidal Hill curve using Sigma Plot (Systat Software Inc, San Jose, California, USA). The LOD was calculated at the minimum TCDD concentration that induced significant dioxin-like activity compared to SC. The LOD was 64 fg TCDD/well which equal 0.427 pg TCDD/mL serum in the present study.

Furthermore, the dioxin-like activity of procedure blank, control serum and test serum samples were calculated as AhR mediated TCDD equivalent (AhR-TEQ) by interpolation of their corresponding AhR activity onto the TCDD titration dose-response to obtain their corresponding AhR-TEQ value (pM). The final net AhR-TEQ of test serum sample was obtained by subtraction of the procedure blank AhR-TEQ value (net AhR-TEQ (pM) = AhR-TEQ _sample_ (pM) − AhR-TEQ _procedure blank_ (pM)). After adjustment to serum lipid data, the final net AhR-TEQ was expressed as pg TEQ/g lipid. For AhR-TEQ of sample being below the AhR-TEQ of procedure blank a value of 0.5 × LOD was assigned [54].

### 2.7. Fetal Growth Indices

The measurements of the newborns were carried out by midwives at birth. We obtained the fetal growth indices including BW, BL, HC and GA at birth from the Chief Medical Office in Greenland. We defined the abnormal BW as follows: low BW, less than 2500 g regardless of GA at the time of birth; macrosomia, when BW exceeded 4500 g [55,56]. Small BL was defined when less than 50 cm and small HC when less than 35 cm [57]. The ponderal index (PI, g/cm^3^) is calculated as [(BW in gram/ (BL in cm)^3^) × 100]. We classified the neonates with PI between 2.2 g/cm^3^ and 3.0 g/cm^3^ as normal [58,59]. Preterm birth was defined as birth before 37 gestational weeks according to the WHO definition [60].

### 2.8. Statistical Analysis

We checked the distribution of data by Q-Q plots. The natural logarithmic transformed data improved normal distribution and homogeneity of the serum AhR-TEQ and POP data, and thus the ln-transformed data were used for the comparison analysis. The AhR-TEQ values evaluated both as continuous and categorized as quartiles. The fetal growth indices were normally distributed and treated as continuous and/or categories.

We used one-way ANOVA to compare AhR-TEQ levels among maternal demographic parameters and categories of fetal growth indices. If differences were observed, multiple comparison ad hoc tests were performed using the least significant difference (LSD) pair wise multiple comparison test for the variables with equal variance (*p* > 0.050), and Dunnett’s T3 test for the variables with an unequal variance (*p* ≤ 0.050). The homogeneity of variance was tested by Levene’s test. Univariate general linear model (ANCOVA) was used to compare the difference under adjustment of covariates/confounders such as age, BMI, n-3/n-6 PUFA ratio, cotinine, education level, alcohol intake during pregnancy and parity. The ANCOVA analysis of PI was further adjusted for GA, since GA is the most important factor of neonatal PI [59].

Spearman correlation analysis was used to evaluate the correlation of AhR-TEQ and characteristics of the study population.

**Figure 3:**
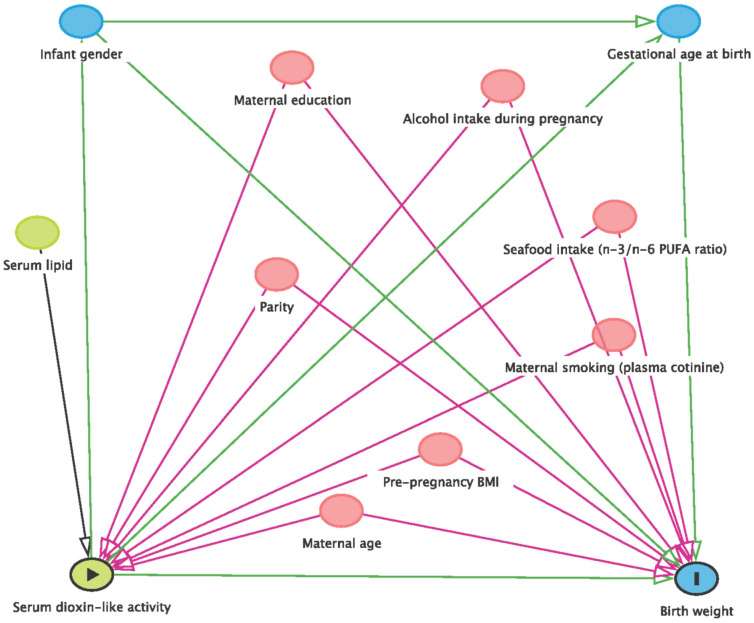
Directed acyclic graph illustrating the potential influences on the relationship between serum dioxin-like activity and birth weight. The figure also applies to other fetal growth indices (birth length, head circumference, ponderal index and gestational age at birth). According to this graph, variables in pink circle were adjusted in the model. The figure was made using dagitty.net [61].

We used multivariable linear regression model with robust standard errors to analyze the association of AhR-TEQ (pg/g lipid) and fetal growth indices with and without adjustment for confounders. We used directed acyclic graph (DAG) principle to identify the confounders (Figure 3) [43,61]. The literature shows that maternal age, BMI, education level, smoking, alcohol intake and parity can influence the fetal growth [62]. In the model, the AhR-TEQ was the independent variable and fetal growth indices were dependent variables. We run both the crude and adjusted model with the selected confounders based on DAG by adjustment of maternal age, pre-pregnancy BMI, maternal education, smoking status during pregnancy represented by plasma cotinine level, alcohol intake during pregnancy and parity. We further adjusted for GA although we are aware of the problem of a potential intermediate [63]. Given that the fatty acids can influence the fetal growth and DLC levels [64,65,66,67], we also further adjusted for seafood intake (n-3/n-6 PUFA ratio) together with other confounders/covariates.

We performed sensitivity analysis in order to explore remaining confounding. In particular, we performed the analyses by stratifying the infant gender due to the gender difference of association between POPs and fetal growth indices [68,69]. Previous study showed that smoking status influence the serum POP induced dioxin-like activity [53,70] and maternal smoking can affect the fetal growth [71,72]. To assess the maternal smoking effect on the association of lipPOP induced dioxin-like activity on fetal growth, in the further analyses we stratified by maternal smoking history. In addition, we restricted the analyses to full term birth (≥37 weeks of gestation) and normal BW (BW 2500–4500 g).

We used linear regression analysis to analyze the association between levels of AhR-TEQ and lipPOPs groups and single DL-PCB and OCP congeners detected in more than 40% samples. Based on previous experience [73], the lipPOP groups included ΣPCB, ΣOCP, ΣlegacyPOP (ΣPCB + ΣOCP) and ΣlipPOP (ΣPCB + ΣOCP + ΣPBDE). The proportion of DL-PCB on total PCB is important to the determined dioxin-like activity (AhR-TEQ), as some PCB congeners such as PCB 28, 128, 138, 153, 170 and 180 elicit antagonistic effect on AhR [74,75] and the AhR activity measured in the present study was the integrated effect of total lipPOP mixture. Thus, the DL-PCBs (PCB105 + PCB118 + PCB156) percentage of total PCB was calculated. We hypothesized that a potential predictor of POP bioaccumulation might also be a potential predictor of serum dioxin-like activity [73]. To avoid over-adjustment, we used the non-lipid adjusted data of POPs (µg/L) and AhR-TEQ (pg/mL) in the linear regression analysis in crude and under adjustment of serum lipid. As known from the literature [62,72], age, BMI, smoking, alcohol intake, seafood intake and parity might influence the serum POP levels. Thus, we used the multivariable linear regression model to assess the relation between the serum non-lipid adjusted dioxin-like activity (pg/mL) and the non-lipid adjusted lipophilic POPs (µg/L) under adjustment for the above lifestyle factors together with serum lipid.

In addition to lipPOPs, the lipophilic metals Hg and Se were measured in whole blood and/or plasma. To evaluate whether these metals contribute to the measured serum dioxin-like activity in pregnant women, the association of serum contaminant levels and AhR-TEQ was further analyzed. Due to a large number of analytes, we used principal component analysis (PCA) [76,77] to identify potential underlying components of POP and metal levels in the blood. PCA group the correlated variables as principal components (PCs) by forming a few linear combinations called “factor loadings”. The factor loading indicate the extent to which the chemical concentrations conformed to the corresponding PCs. The lipPOPs (µg/L) and metals (µg/L) with detectable percentage of more than 40%, were included into the model: seven OCPs (p,p’-DDE, β-HCH, HCB, Oxychlordane, trans-Nonachlor, cis-Nonachlor and Mirex), ten PCBs (PCB 105, 118, 138, 153, 156, 170, 180, 183, 187 and 99) and two fatty metals (Hg and Se). The number of PCs were extracted on eigenvalues (>1) and scree plot. We used varimax rotation to obtain a set of independent and best interpretable components [77]. The Kaiser–Mayer–Olkin (KMO) test (0.908) and the Bartlett test of sphericity (*p* < 0.0001) were used to test suitability of the analysis. All factor loadings >0.4 were used to identify the variables comprising a principal component. The multiple linear regression analysis of the association between the identified PCs and the serum AhR-TEQ was similar with the lipPOP congeners mentioned earlier.

The linear regression analysis was performed by simple linear regression (i.e., the variables were not ln-transformed) and ln-linear regression analysis (i.e., the variables were ln-transformed). The assumptions of linear regression regarding normality, linearity, homoscedasticity, absence of multi-collinearity were checked by normal predicted probability plot, scatterplot of the residuals, and variance inflation factor values. We tested the autocorrelation using the Durbin–Watson test. The simple- and ln-linear linear regression models had similar fit of the assumption of linear regression, thus we report the results of simple linear regression analysis of AhR-TEQ, fetal growth indices and lipPOPs.

Due to a certain amount of missing data, we also performed multiple imputation, which allows including participants with incomplete data set in the statistical analysis. Multiple imputations were performed for variables with >5% missing values, assuming the missing values to be missed at random [78,79]. The multiple imputation created multiple different data sets (m > 1) with missing values replaced by random plausible values based on the participant’s characteristics in the complete data set.

The statistical analyses were performed in SPSS 27.0 (IBM Inc., Chicago, IL, USA). The term statistically significant level denotes a *p*-value ≤ 0.050 and for a *p*-value ≤ 0.080 as borderline significant.

## 3. Results

### 3.1. The Serum Levels of the Combined DL-PCBs and LipPOP Induced Dioxin-like Activity (AhR-TEQ)

Table 1 shows the DL-PCB congener (PCB 105, 118 and 156) were detected in 44.4%, 98.6% and 80.4% of the serum samples from the ACCEPT pregnant women, respectively. We found a significant regional difference of DL-PCBs and lipPOP levels, with East and North having higher levels. However, the percentage of sum DL-PCB respective to the sum of all PCB (ΣDL-PCB/ΣPCB) in East region was significantly lower compared to other regions (Table 1).

We detected significant dioxin-like activity (AhR-TEQ pg/mL serum) in 87.7% serum samples of the Greenlandic Inuit pregnant women. The median level of AhR-TEQ was 1.32 pg/mL serum and 86.2 pg/g serum lipid (Table 1). We found no significant regional difference, although a tendency of lower lipid adjusted AhR-TEQ (pg/g serum lipid) found for the East region, which may relate to the significantly higher serum lipid level and lower proportion of ΣDL-PCB respective to the ΣPCB in East region (Table 1).

Table 2 shows the distribution of AhR-TEQ in relation to the characteristics of the included ACCEPT pregnant women. The median age of the pregnant women was 27 years, and around 24% were overweight (25 < BMI ≤ 30 kg/m^2^) and 16% were obese (BMI > 30 kg/m^2^). Most participants had a primary school (37.9%) and technical college (28.5%) education. The frequency of women smoking either before pregnancy or during pregnancy was about 37%. Before pregnancy, 45% of the participants reported consuming alcohol less than one time a month while around 48 % reported consuming alcohol at least one time a month. Around 2.0% participants still consumed alcohol during pregnancy at least once a month. About 39% women were nulliparous, while most women had given birth of at least one child (Table 2).

As shown in Table 3, most infants (92.8%) were in the normal BW range while less than 4% had either low BW (<2500 g) or macrosomal (>4500 g). There were 17.8% infants having a BL less than 50 cm. According to the PI classification, 89.3% infants were in the normal range (2.2–3.0 g/cm^3^), while 10.5 % infants had abnormal PI value. The HC of 44.3% infants was less than 35 cm. Most infants were born full term (GA ≥ 37 weeks) while around 5% were born preterm. More than half (52.9%) of the infants were male (Table 3). Male infants had significantly higher BW, BL and HC than female infants (Table S1).

Table 3 also shows the results of maternal serum AhR-TEQ in relation to the fetal growth indices. The median level of serum AhR-TEQ in the pregnant women giving birth to infants with normal BW were slightly lower than those with low BW (<2500 g) or macrosomia infants (BW > 4500 g). After adjustment of maternal age, BMI, plasma cotinine, n-3/n-6 PUFA ratio, alcohol intake during pregnancy, education level and parity, this difference was borderline significant (Table 3, *p* = 0.07). We observed significant (*p* = 0.05) higher level of serum AhR-TEQ for the women having infants with BL shorter than 50 cm after adjustment of confounders (Table 3). We found significantly lower maternal serum level of lipid adjusted AhR-TEQ for infants born preterm than full term infants, although the significance disappeared after confounder adjustment (Table 3).

We did not observe any differences in maternal serum AhR-TEQ level among the PI groups or the HC groups (Table 3). No significant differences of serum levels POPs and AhR-TEQ in women giving birth of male and female infants were observed (Table S1).

### 3.2. Associations between Dioxin-like Activity and Lipophilic POPs

As shown in the Table S2, PCB118, PCB138, PCB153, PCB156, PCB170, PCB180, PCB 183, PCB187, PCB 99, HCB, Oxychlordane, p,p’-DDE, β-HCH, trans-Nonachlor, cis-Nonachlor and Mirex were detected in more than 50% and PCB105, PCB128 and p,p’-DDT in 44.4%, 15.6% and 15.7% of serum samples, respectively. The PBDEs were only above the LOD in up to 4.44% of serum samples. PCB52, γ-Chlordane, Aldrin, PBDE15, PBDE17, PBDE25, PBDE28 and PBDE33 were not detectable. We detected Hg in 78.6% of whole blood, and Se in all whole blood samples and 99.8% of plasma sample, respectively (Table S2).

Table 4 shows the linear regression analyses of the association between serum lipPOPs and AhR-TEQ. In general, we observed non-significant positive associations of AhR-TEQ and the individual lipPOP congeners. After adjustment for maternal age, pre-pregnancy BMI, plasma cotinine, n-3/n-6 PUFA ratio, alcohol intake during pregnancy, parity and serum lipid, the positive association between AhR-TEQ and PCB156 was significant (*p* = 0.02), showing that an increase of 1 µg/L PCB156 relates to an increase of 6.59 pg/ml AhR-TEQ. We observed a borderline association of AhR-TEQ and Oxychlordane (Table 4). The association of AhR-TEQ with the ratio of ΣDL-PCB to ΣPCB (ΣDL-PCB/ΣPCB) seemed to be high (β = 9.93, *p* = 0.11), although non-significant. However, the association of AhR-TEQ and ΣPCB, ΣOCP, Σlegacy POP and Σ lipPOP were close to zero (β < 0.1, Table 4).

We used PCA analysis to identify potential principal components (PCs) of POPs and metal levels in the blood and identified two distinct PCs. The first and most distinct principal component (PC-1) mainly had high factor loadings for several PCBs and OCPs representing the lipPOPs explained 75.53% of the total variation in the original chemical concentrations (Table S3). The second component (PC-2) had high factor loadings for metals (Hg and Se) and explained 11.33% of the total variation in the original chemical concentrations (Table S3). As shown in Table 4, the linear regression analyses showed positive tendency of AhR-TEQ to the identified lipophilic POPs component, PC-1 (adjusted β = 0.31, *p* = 0.17) while the linear regression coefficient of AhR-TEQ with lipid soluble metal component, PC-2, was close to zero (Table 4).

### 3.3. Correlation of Maternal Characteristics with Fetal Growth Indices and Serum Dioxin-like Activity

Table 5 presents the Spearman correlation coefficients of parameters. Pre-pregnancy BMI was significantly positive correlated with BW, BL, PI and HC, while inversely correlated with AhR-TEQ level. The smoking biomarker cotinine and self-reported smoking history correlated significant inversely with BW, BL, HC and GA. Seafood intake (n-3/n-6 PUFA ratio) was significantly positive correlated to GA and lipid adjusted AhR-TEQ. Alcohol intake during pregnancy and HC was significant inversely correlated with HC. Moreover, parity correlated significant inversely with lipid adjusted AhR-TEQ. We observed a positive significant correlation between maternal education level and BW, BL and HC. The GA correlated significantly with BW, BL, PI and HC. In contrast to BMI, seafood intake and parity, we found no significant correlation between the AhR-TEQ level and maternal age, plasma cotinine, smoking history, alcohol intake, maternal education and GA (Table 5).

### 3.4. Association of the Combined Maternal Serum Dioxin-like Activity and Fetal Growth Indices

Because the results using non-lipid adjusted AhR-TEQ (pg/mL) were similar with that using lipid adjusted AhR-TEQ (pg/g lipid), we present the associations of maternal dioxin-like activity with fetal growth indices using the lipid adjusted AhR-TEQ (pg/g lipid). There was a non-significant inversely associations between continuous AhR-TEQ and BW in both the crude data and the adjusted data, whereas the associations between continuous AhR-TEQ and BL, PI, HC and GA were almost null (Table S4).

Grouping the serum AhR-TEQ into quartiles showed the third quartile of AhR-TEQ being borderline positively associated with PI (adjusted β_Q3_ = 0.13, *p* = 0.054, Figure S1C). However, for all fetal growth indices we observed no significant trends (*p* for trend > 0.050, Figure S1).

### 3.5. Sensitivity Analysis of Association between AhR-TEQ and Fetal Growth Indices

The associations of stratifying by the offspring gender (Table S5) shows similar patterns as for the pooled gender data (Table S4 and Figure S1). However, for male infants, the AhR-TEQ quartiles inversely associated with BL borderline (*p* trend = 0.08, Table S5). The third quartile of AhR-TEQ positively associated with PI (*p* = 0.01) and GA (*p* = 0.05). For the female infants, there were no significant associations of AhR-TEQ and fetal growth indices (Table S5).

**Figure 4:**
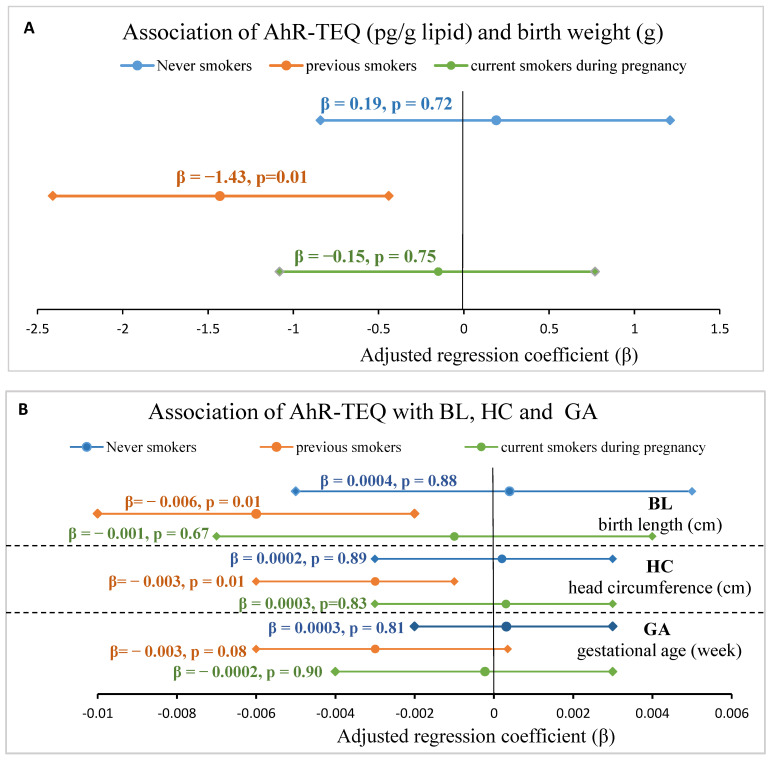
The association of continuous AhR-TEQ (pg/g lipid) and fetal growth indices stratified by maternal smoking history. (**A**) Association of AhR-TEQ and birth weight (g). (**B**) Association of AhR-TEQ and birth length (cm), head circumference (cm) and gestational age (week). The given regression coefficient was adjusted for maternal age, pre-pregnancy BMI, alcohol consumption during pregnancy and parity. Round circle: estimate (β). Diamond circle: end of 95% CI.

Figure 4 shows the associations of continuous AhR-TEQ and growth indices by stratifying the maternal smoking history. For previous smokers, the AhR-TEQ significant inversely associated with BW (Figure 4A), BL and HC while borderline with GA (Figure 4B) upon adjustment for maternal age, pre-pregnancy BMI, alcohol consumption during pregnancy, education level and parity. No significant associations were observed for never smokers and current smokers during pregnancy (Figure 4). No significant association between AhR-TEQ and continuous PI was observed for the different smoking history (Table S6).

We also analyzed AhR-TEQ quartiles stratified by smoking history (Table S6, gives also the continuous data). In never smoking women, the third quartile of AhR-TEQ significantly associated with PI (adjusted β_Q3_ = 0.17, *p* = 0.03) after covariates adjustment. Upon further adjustment of GA, this significant association sustained (β_Q3_ = 0.19, *p* = 0.02). For previous smokers, we found no significant data for the adjusted data, but unadjusted the AhR-TEQ positively associated with BL at the second quartile (β_Q2_ = 0.94, *p* = 0.05) and GA at the third quartile (β_Q3_ = 0.69, *p* = 0.02) (Table S6). We found no significant associations of AhR-TEQ quartiles and fetal growth for current smokers during pregnancy (Table S6).

Cotinine is an active metabolite of nicotine and used as a biomarker to detect current tobacco smoke exposure. Considering that the self-report smoking status might result in bias, even though we generally find good agreement between smoking status and cotinine levels, we further categorized smoking status using the cut-off level for plasma cotinine (0.5 ng/mL) and similar results were observed (data not shown).

Restricting the analyses to women giving birth full term (gestational age > 37 weeks), there was an inversely significant association of the continuous AhR-TEQ with BW (β = −0.42, *p* = 0.04) and borderline significant with BL (β = −0.002, *p* = 0.07) for the crude data. However, after adjustment of confounders/covariates, the significance disappeared (Table S7). No significant associations between AhR-TEQ quartiles and fetal growth indices were observed. For the preterm birth group (GA < 37 weeks), no significant association between continuous AhR-TEQ and fatal growth indices were observed which might be due to the very small sample size (Table S7).

We further performed the multiple regression analysis for the association between AhR-TEQ and fetal growth indices with further adjustment of seafood intake (n-3/n-6 PUFA ratio). We obtained similar results with mildly attenuated associations (data not shown).

Due to a certain amount of missing data, the above analyses were also performed for the multiple simulation data and similar results were obtained (data not shown).

## 4. Discussion

The present study measured the serum level of combined dioxin-like activity (AhR-TEQ) induced by the lipPOP mixtures in Inuit pregnant women from five Greenlandic regions, assessing their association to fetal growth indices including BW, BL, PI, HC and GA. We observed significantly levels of AhR-TEQ in more than 87% of serum sample with the median level of 86.2 pg TEQ/g lipid. There was no significant difference in serum AhR-TEQ among the five Greenlandic regions. Serum AhR-TEQ non-significantly positive associated with serum levels of DL-PCBs, indicating the measured AhR-TEQ reflect the burden of dioxins and DLCs in the study population.

We observed AhR-TEQ correlated negatively with pre-pregnancy BMI, while positively with seafood intake (serum n-3/n-6 PUFA ratio). The nulliparous women had significantly higher serum AhR-TEQ level and the AhR-TEQ level decreased with increasing parity.

Maternal serum AhR-TEQ tended to associate inversely with fetal growth indices, and these associations seem to be slightly stronger in male offspring. Among the previous smokers, the associations of maternal serum AhR-TEQ and BW, BL and HC were stronger and significant compared to never smokers and current smokers during pregnancy, suggesting the maternal smoking history might modify the associations.

### 4.1. Levels of Serum Dioxin-like Activity (AhR-TEQ)

The dioxin-like activity determined by the AhR-reporter gene bioassay has been proven to provide a global measure of AhR-mediated activity in biological samples that contain complex mixtures of compounds with different affinity and efficacy for the AhR [25,52,53,70]. In the present study, the median serum level of dioxin-like activity was 86.2 pg TEQ/g lipid in the Greenlandic Inuit pregnant women with median age of 27 years. Their AhR-TEQ level was much lower than the level of Greenlandic non-pregnant women recruited in 1997-1998 (152 pg TEQ/g lipid) [80] and in 1999–2005 (167 pg TEQ/g lipid) [53]. The Greenlandic Inuit women in these previous studies were older (median age 35-37 years old) [53,80] than the present study (27 years old). Owing to their very long half-lives, lipPOPs accumulate in the body throughout the life, so in a given population older people have a higher body burden of dioxin-like compounds. Given the positive correlation between age and body burden of POPs including serum dioxin-like activity [81,82,83], the lower dioxin-like activity of pregnant Inuit women in the present study might be related their younger age as well as a decreasing trend of POPs in the Arctic due to regulations [5,45,84]. The declining trend of POPs levels is evidenced by the fact that in the present study the sum of the levels of 14 PCBs of Greenlandic pregnant women enrolled during 2010–2015 was 181 µg/kg lipid while in Greenlandic women recruited during 1999–2005 the level was 636 µg/kg lipid [53]. The median percentage of DL-PCB relative to total sum PCB (ΣDL-PCB/ΣPCB) was 7.40% in the present study and lower than in 1999–2005 (9%) [53]. This also partly explains the lower AhR-TEQ level in the present study. The global decrease in other DLCs (e.g., PCDD/Fs, other DL-PCBs) because of the regulation strategy [85] may also play role in the decreased AhR-TEQ, but it is difficult to further discuss this since other DLCs were not measured in the ACCEPT pregnant women. Compared to the reported AhR-TEQ levels of Nunavik Inuit adults from Canada (geometric mean 8.9 pg/g lipid) [83] and European populations including Danish (geometric mean 15.5–46.4 pg/g lipid) [43,86], the serum AhR-TEQ in the present study is higher. This may be explained by differences in lifestyle and dietary habits [54], together with difference in protocol, cell line and exposure time as discussed previously [53,83].

Although, we did not find any significant regional difference of serum AhR-TEQ, the lipid adjusted AhR-TEQ level was lowest in East. This can be due to significantly higher serum lipid level and lower ratio of ΣDL-PCB/ΣPCB in East region.

### 4.2. Determinants of Serum Dioxin-like Activity (AhR-TEQ)

Similar with a previous study of Nunavik Inuit adults in Canada, we observed in the present study that the marine food intake (n-3/n-6 PUFA ratio) positively correlated with serum dioxin-like activity, indicating that marine food is one source of DLCs exposure [83]. One study showed that n-3 PUFA supplements derived from different marine mammals and fish elicited AhR activity, and the AhR activity was high in supplements derived from salmon and tuna [87]. Salmon is one of the traditional foods in Greenland and may contribute to the positive association of AhR-TEQ and n-3/n-6 PUFA ratio.

We found no obvious correlation between age and AhR-TEQ in the present study, which might be because of the narrow age range of the participants (18–42 years), and only 5% were above 35 years old.

The AhR-TEQ correlated negatively with pre-pregnancy BMI, supported by the fact that the DLCs are stored in the adipose tissues and released from adipose tissue during weight loss [88,89,90]. The negative correlation between parity and AhR-TEQ indicate that the mother passes the DLCs to the fetus and the child during breastfeeding due to their lipophilic property.

### 4.3. Associations of Maternal AhR-TEQ with Fetal Growth Indices

Reports have shown that pregnant women exposed to high level of dioxins and DLCs affect fetal growth [37,91,92]. The 40 years follow-up study of women exposed to high levels of TCDD from an industrial explosion in Seveso, Italy observed a non-significantly inverse association between maternal TCDD exposure and infant BW for those with first pregnancy after accident [93]. However, there are conflicting results regarding the effect of low level DLCs exposure on fetal growth. Some studies showed that low levels of DLCs in pregnant women associated with decreased fetal growth indices [32,41,94], while others reported no associations or a potential inverse association [39,93,95]. A Danish study showed a trans-placental transport of DLCs measured by the AhR reporter gene bioassay [86]. Few studies measured the AhR activity of DLC mixtures using reporter gene assay and explored its association with fetal growth, and the results were inconsistent. Vafeiadi et al. reported that across the European population including Denmark, Greece, Norway, Spain and England, cord blood AhR-TEQ associated significant inversely with GA and non-significantly with BW [43]. However, the study found no associations between the AhR-TEQ in maternal blood drawn at delivery and any fetal growth indices [43]. Similar result was shown in a Danish study with 77 participants and maternal blood taken during Weeks 8 to 25 of gestation [42].

In the present study, we observed that maternal serum AhR-TEQ weakly and inversely associated with fetal growth indices in Greenlandic Inuit. The association of AhR-TEQ and fetal growth indices seemed slightly stronger in male offspring. Similar to our result, the retrospective study in Yusho, Japan showed that maternal exposure to high levels of dioxins and DL-PCBs significantly decreased BW among male infants, but not female infants [37]. This sex-specific result is supported by other studies and suggest a greater susceptibility of male infants to the AhR activating contaminants, either in the embryonic or in the fetal period [38,40,43,96,97].

A study reported that high level of some DLCs such as 2,3,4,7,8-pentachlorodibenzofuran and PCB169 in women from Yusho, Japan associated with preterm birth [98]. Whereas no significant association between TCDD exposure and preterm birth was observed for highly exposed population in Seveso, Italy over 30 years follow-up [99]. Using logistic regression analysis, we did not find any association between AhR-TEQ in the pregnant women and preterm birth (data not shown). Since our study only included 24 offspring born preterm, the power of the statistical analysis was low. The discrepancies in the results could be due to different levels of exposure, variation in the mixtures of toxic compounds in different settings, differences in the study design, population characteristics, sample sizes, and timing of sample measured.

Studies have shown significant trans-placental transfer of environmental tobacco smoke constituents including PAHs from mother to fetus [100], and cigarette smoke both before and during pregnancy inversely associated with fetal growth with stronger association found in women smoking during pregnancy [72,101]. In the present study, we observed the maternal AhR-TEQ significant inversely associated with fetal growth indices in previous smokers and non-significantly in mothers smoking during pregnancy. Studies have shown that tobacco smoke contains substances including dioxins and DLCs and elicited high dioxin-like potential [102,103]. However, smoking history or plasma cotinine were not correlated with AhR-TEQ in the present study, even though we observed the lowest AhR-TEQ in never smokers (74.4 pg/g lipid) and higher in current smokers during pregnancy (88.8 pg/g lipid) and previous smokers (89.3 pg/g lipid). Interestingly, before lipid adjustment, the AhR-TEQ were the lowest in previous smokers (1.14 pg/mL), slightly higher in never smokers (1.28 pg/mL) and current smokers during pregnancy (1.38 pg/mL). As the serum lipid level was the lowest in previous smokers (6.60 g/L) followed by current smokers during pregnancy (7.50 g/L) and never smokers (8.10 g/L), the association of AhR-TEQ and fetal growth indices of different smoking history might be affected via influencing the lipid adjusted AhR-TEQ level. Nevertheless, by using non-lipid adjusted AhR-TEQ in the analysis, similar association pattern as in the analysis with lipid adjusted data (shown in Figure 4 and Table S6) were seen among smoking history groups (data not shown).

Fierens et al. reported that the serum dioxin levels were significantly decreased in female current smokers [104]. They speculated this might involve the cross-talk between AhR and ER mediated signaling pathways and that the cooperation between AhR and ER could lead to a synergistic potentiation of dioxin metabolism by compounds in the tobacco smoke [103]. In addition, the estrogen level increase significantly during pregnancy and the synergistic effect of AhR and ER on DLCs metabolism would be stronger in previous smokers. Hence, the effect of prenatal exposure to DLCs on the fetal growth in women smoking during pregnancy might be weaker than in previous smokers [103]. We have no information about smoking duration, amount of cigarette consumption, passive smoking or when the previous smokers stopped smoking, thus the influence of these factors on the association of maternal serum AhR-TEQ and fetal growth in the previous and current smoking women need more research to elucidate.

A study measured the DL- PCBs and PCDD/Fs in maternal blood, cord blood and cord tissue in 41 Japanese mother-infant pairs and showed that PCB105, 118 and 156 can be transferred through the placenta and accumulate in the cord tissue/blood [105]. A previous Chinese study reported umbilical cord blood level of PCB118 associated with smaller BL and HC [106]. A recent US Fetal Growth Studies–Singleton cohort study [107] recruited non-obese, low-risk pregnant women of different race/ethnic before gestation week 14 during 2009–2019. They observed DL-PCBs and OCPs were negatively associated with HC, and the POP mixtures elicited stronger effect than individual chemicals. This observation may reflect the differences in risk owing to the complex interactions among individual chemicals and supports the association of AhR-TEQ and fetal growth indices, as AhR-TEQ is an integrated biomarker of dioxins and DLC mixtures [70]. In the present study, the measured PCB 105, 118 and 156 seemed positively associated with the measured AhR-TEQ, suggesting that they were contributors to the measured dioxin-like activity. Previous studies showed non-DL PCB such as PCB 28, 128, 138, 153, 170 and 180 elicit antagonistic effect on AhR [74]. Our unpublished data also showed that non-DL PCBs seemed to inhibit the DL-PCB induced AhR activity and thus the ratio of DL-PCBs to sum PCBs affected the determined combined AhR-TEQ. This may partly explain the inverse association of AhR-TEQ and fetal growth indices was not statistically significant.

The biological mechanism for the effect of POPs on birth size is not well established. Animal studies suggested potential immune-toxic effects of dioxin-like PCBs [108] and impaired immune function during pregnancy maybe associated with fetal growth in humans [109]. Cross-talks between AhR agonists such as DLCs and hormone receptors such as ER and thyroid hormone receptor (TR) have been observed [110,111]. POPs can disrupt and reduce levels of circulating thyroid hormones (THs) [112] and hypothyroidism has been shown to be associated with fetal growth [113]. Dioxins and THs share common molecular reactivity properties and possibly similar molecular recognition in biochemical systems [114]. A study suggested that dioxin-like PCBs elicited antagonist TR activity [115]. The findings of a recent US cohort [107] suggested that the fetuses of Asian women were more sensitive to higher-chlorinated PCBs compared with fetuses of non-Asian women, resulting in smaller femur length which is a predictor of BL [116]. This may partly explain the association of AhR-TEQ and BL in the present study due to similar genetic background between Inuit and Asians [117]. Estrogens can also affect the fetal growth by multiple mechanisms [118,119]. Epidemiologic studies have shown positive associations of maternal serum levels of estrogens and birth size such as BW and BL [120,121]. The AhR-mediated inhibitory AhR-ER cross-talk have been reported [15,122] and thus can affect estrogen homeostasis. The anti-estrogenic effect of AhR ligands may also explain the observed inverse association of AhR-TEQ and fetal growth indices.

### 4.4. Strength and Limitation

To our knowledge, the present study is the first study exploring the association of maternal level of combined dioxin-like activity induced by lipPOPs and fetal growth in Greenlandic Inuit. Serum dioxin-like activity (AhR-TEQ) derived from ex vivo reporter gene bioassay represents the combined induced effect for the AhR of complex mixture. This assay cannot distinguish between the effects of the specific congeners. Studies indicated that PCDDs influenced decrease in BW more than PCDFs and PCBs, as PCDDs transfer more readily than PCDFs and PCBs from maternal blood to the fetus through the placental transfer by passive diffusion [37,123]. Compared to the TEQ value obtained from GC-MS measurement (median: 0.42 pg/g lipid), the AhR-TEQ level obtained from AhR reporter gene bioassay (median: 86.2 pg/g lipid) is much higher. This is expected because TEQ value obtained from AhR bioassay integrates all activities and possible interactions of all individual congeners in the mixture. In the present study, we only measured levels of three DL-PCBs and have no information on the levels of PCDD/Fs and other DL-PCBs. Hence, we cannot evaluate the contribution of these specific congeners for the measured AhR-TEQ and their associations to fetal growth indices. However, a previous Canadian study indicated that TEQ were mostly accounted for by PCBs (3-fold higher DL-PCB derived TEQ than TEQ derived from PCDD/Fs) in the Inuit male and female population [124]. In the present study, we detected the three DL-PCBs in 44–99% of the included samples from Greenlandic Inuit pregnant women and they may play the major role in the AhR-TEQ and fetal growth indices association. However, the contribution by other DL-PCBs and PCDD/Fs might influence the data. In future studies measurement of dioxins and PCDFs might elucidate the influence of other dioxin and furans.

Although the blood samples were taken early in pregnancy, the persistent nature of lipPOPs suggested that it is a reasonable reflection of exposure during the complete prenatal period. Exposure misclassification may be reduced by measurement of specific receptor activations through the in vitro AhR reporter gene bioassay as variation in uptake and affinity to the AhR are integrated in the toxic potency measurements [125]. Multiple interactions among co-exposures, their metabolites and endogenous AhR activating compounds/molecular should also be taken into consideration [70].

## 5. Conclusions

Pregnant Inuit women in the Greenland are exposed to the lipPOPs through their traditional mammal marine food intake. The lipPOP induced dioxin-like activity mediated by the AhR, the lifestyle and reproductive factors can influence the level of serum dioxin-like activity. Prenatal exposure to dioxins and DLCs can influence on the fetal growth. Smoking history might modify the association between dioxin-like activity of DLC mixtures and fetal growth, and this needs further studies.

## Figures and Tables

**Table 1:** Serum levels of lipophilic POPs and AhR-TEQ in ACCEPT pregnant women.

			North	Disko Bay	West	South	East	*p*	All Greenland Regions
**POPs (µg/kg serum lipid)**	% over LOD	n	29	104	255	41	18		447
PCB105	44.4%	Mean ± SD	3.89 ± 3.34	2.13 ± 1.52	1.75 ± 1.77	1.54 ± 1.02	11.9 ± 9.78	**<0.0001**	2.37 ± 3.29
		Median (p25–p75)	3.00 (1.95–4.95)	1.85 (1.95–3.28)	1.00 (1.00–2.20)	1.00 (0.50–2.10)	9.30 (4.38–16.5)		1.60 (1.00–2.60)
PCB118	98.6%	Mean ± SD	18.9 ± 15.5	12.0 ± 7.55	9.93 ± 9.87	9.01 ± 4.84	68.2 ± 66.8	**<0.0001**	13.2 ± 19.7
		Median (p25–p75)	14.0 (8.90–25.5)	9.60(6.03–17.0)	7.30 (4.80–12.0)	8.80 (4.15–12.0)	45.0 (23.0–82.8)		9.00 (5.40–14.0)
PCB156	80.4%	Mean ± SD	7.05 ± 13.5	3.72 ± 2.74	4.06 ± 4.04	3.90 ± 2.52	16.7 ± 15.1	**<0.0001**	4.67 ± 6.20
		Median (p25–p75)	4.60 (2.30–6.35)	3.00 (1.90–5.00)	3.00 (1.80–4.90)	3.10 (1.95–5.45)	11.5 (7.15–19.5)		3.20 (2.00–5.40)
∑DL–PCB		Mean ± SD	29.8 ± 28.9	17.8 ± 11.1	15.7 ± 14.4	14.4 ± 7.20	96.8 ± 90.2	**<0.0001**	20.3 ± 27.8
		Median (p25–p75)	20.1 (14.2–36.7)	15.4 (8.85–25.0)	11.9 (7.60–18.9)	14.3 (7.24–18.6)	69.1 (34.1–117)		13.9 (8.40–21.9)
∑PCB		Mean ± SD	378 ± 489	218 ± 125	212 ± 219	225 ± 108	1821 ± 1981	**<0.0001**	290 ± 545
		Median (p25–p75)	257 (168–397)	188 (116–283)	157 (104–237)	199 (149–313)	1181 (603–1857)		181 (116–282)
∑DL–PCB/∑PCB (%)		Mean ± SD	8.33 ± 1.75	8.05 ± 1.72	7.56 ± 1.77	6.55 ± 1.74	6.01 ± 1.45	**<0.0001**	7.57 ± 1.82
		Median (p25–p75)	8.53 (7.01–9.28)	8.19 (6.47–9.17)	7.42 (6.38–8.76)	6.48 (5.38–7.32)	5.96 (4.70–7.46)		7.41 (6.35–8.88)
∑OCP		Mean ± SD	525 ± 416	315 ± 196	267 ± 307	279 ± 153	2781 ± 3493	**<0.0001**	397 ± 885
		Median (p25–p75)	416 (266–642)	271 (161–434)	198 (127–304)	256 (160–390)	1501 (827–2779)		232 (143–401)
∑Legacy POPs		Mean ± SD	903 ± 857	533 ± 314	480 ± 520	504 ± 253	4624 ± 5473	**<0.0001**	688 ± 1422
		Median (p25–p75)	674 (452–1024)	449 (283–713)	361 (238–529)	480 (307–709)	2764 (1390–4652)		422 (265–686)
∑Lip POPs		Mean ± SD	920 ± 855.	551 ± 314	497 ± 519	522 ± 253	4552 ± 5328	**<0.0001**	705 ± 1423
		Median (p25–p75)	693 (465–1049)	462 (294–725)	373 (256–549)	504 (334–732)	2866 (1403–4676)		434 (280–695)
**Dioxin–like activity**									
AhR–TEQ (pg/mL serum)	87.7%	n	29	107	259	43	18	0.65	456
		Mean ± SD	2.05 ± 2.00	1.53 ± 1.29	1.79 ± 1.72	1.64 ± 1.28	1.49 ± 1.52		1.72 ± 1.60
		Median (p25–p75)	1.67 (0.52–2.90)	1.29 (0.42–2.22)	1.32 (0.65–2.41)	1.32 (0.60–2.62)	1.38 (0.21–2.17)		1.32 (0.56–2.40)
AhR–TEQ (pg/g serum lipid)	87.7%	n	29	104	255	41	18	0.30	447
		Mean ± SD	154 ± 194	105 ± 101	125 ± 123	118 ± 101	71.9 ± 65.8		119 ± 121
		Median (p25–p75)	98.2 (36.1–197)	76.8 (24.2–147)	90.4 (42.5–163)	80.1 (35.7–180)	58.8 (15.8–101)		86.2 (35.1–160)
**Serum lipid (g/L serum)**		n	29	104	255	41	18	**0.05**	447
		Mean ± SD	7.44 ± 1.80	7.47 ± 1.92	7.40 ± 1.79	7.37 ± 1.67	8.76 ± 1.66		7.47 ± 1.82
		Median (p25–p75)	7.60 (5.85–9.15)	7.10 (6.20–8.10)	7.30 (6.00–8.50)	6.90 (6.10–8.50)	8.70 (7.75–10.0)		7.30 (6.10–8.60)

LOD: limit of detection; n: total number of participants having information for the parameter. p25: 25th percentile, p75: 75th percentile. *p* value was calculated with one-way ANOVA analysis on ln-transformed data. Bold value indicates statistical significance (*p* < 0.050). DL-PCB (dioxin-like PCB) included PCB105, PCB118 and PCB156; ΣLegacy POPs included 14 PCBs and 11 OCPs, ΣLip POP: lipophilic POPs including 14 PCBs, 11 OCPs and 9 PBDEs and 1 PBB. AhR-TEQ: aryl hydrocarbon receptor mediated TCDD toxic equivalent.

**Table 2:** ACCEPT pregnan women characteristics and maternal serum AhR–TEQ.

		AhR–TEQ (pg/mL)			AhR–TEQ (pg/g lipid)		
		n (%)	Mean ± SD	Median (p25–p75)	n	Mean ± SD	Median (p25–p75)
All		456 (100)	1.72 ± 1.60	1.32 (0.56–2.40)	447 (100)	119 ± 121	86.2 (35.1–160)
Maternal age (years)	≤27	239 (52.4)	1.72 ± 1.63	1.35 (0.52–2.33)	232 (51.9)	123 ± 130	86.6 (32.9–163)
	>27	217 (47.6)	1.71 ± 1.57	1.30 (0.56–2.51)	215 (48.1)	115 ± 110	85.9 (35.8–157)
	Missing	0 (0.00)			0 (0.00)		
	*p value*	*0.99*			*0.84*		
	*Adjusted p value ^a^*	*0.55*			*0.53*		
Pre–pregnancy BMI (kg/m^2^)	Underweight (<18.5)	12 (2.63)	2.00 ± 2.30	1.37 (0.45–2.47)	12 (2.68)	181 ± 261	80.7 (27.9–254)
	Normal weight (18.5–24.9)	246 (53.9)	1.87 ± 1.75	1.38 (0.68–2.56)	238 (53.2)	133 ± 130	91.6 (43.2–178)
	Overweight (25–30)	111 (24.3)	1.48 ± 1.30	1.17 (0.44–1.99)	110 (24.6)	99.9 ± 95.3	70.3 (25.8–134)
	Obese (>30)	73 (16.1)	1.48 ± 1.31	1.17 (0.27–2.13)	73 (16.3)	95.9 ± 81.4	85.0 (20.7–136)
	Missing	14 (3.07)			14 (3.13)		
	*p value*	*0.10*			*0.06 ^#^*		
	*Adjusted p value ^b^*	*0.35*			*0.31*		
Maternal education	Primary school	173 (37.9)	1.63 ± 1.47	1.24 (0.56–2.35)	166 (37.1)	113 ± 121	79.7 (34.5–157)
	High school	74 (16.2)	1.79 ± 1.76	1.36 (0.32–2.54)	73 (16.3)	127 ± 141	90.5 (23.6–159)
	Technical college	130 (28.5)	1.84 ± 1.85	1.37 (0.59–2.47)	129 (28.9)	124 ± 122	90.4 (39.9–165)
	University	78 (17.1)	1.67 ± 1.26	1.56 (0.59–2.52)	78 (17.4)	118 ± 97.4	105 (38.7–165)
	Missing	1 (0.22)			1(0.22)		
	*p value*	*0.95*			*0.95*		
	*Adjusted p value ^c^*	*0.85*			*0.91*		
Maternal smoking history	Never smokers	114 (25.0)	1.74 ± 1.60	1.28 (0.51–2.39)	111 (24.8)	112 ± 122	74.4 (25.8–139)
	Previous smokers	173 (37.9)	1.56 ± 1.38	1.14 (0.48–2.33)	172 (38.5)	120 ± 115	89.3 (31.5–163)
	Current smokers during pregnancy	168 (36.8)	1.86 ± 1.79	1.38 (0.65–2.50)	163 (36.5)	123 ± 127	88.8 (40.6–165)
	Missing	1 (0.22)			1 (0.22)		
	*p value*	*0.28*			*0.33*		
	*Adjusted p value ^d^*	*0.51*			*0.33*		
Maternal alcohol intake before pregnancy	<1 time a month	205 (45.0)	1.65 ± 1.39	1.30 (0.61–2.39)	201 (45.0)	115 ± 107	84.7 (40.9–161)
	1 time a month	71 (15.6)	1.70 ± 1.52	1.35 (0.63–2.51)	71 (15.9)	115 ± 116	92.5 (37.6–144)
	2–3 times a month	95 (20.8)	1.98 ± 2.02	1.55 (0.52–2.64)	93 (20.8)	132 ± 134	92.5 (32.9–175)
	≥1 time a week	51 (11.2)	1.63 ± 1.75	1.14 (0.55–1.88)	48 (10.7)	115 ± 162	65.7 (23.2–133)
	Missing	34 (7.46)			34 (8.50)		
	*p value*	*0.73*			*0.68*		
	*Adjusted p value ^e^*	*0.66*			*0.58*		
Maternal alcohol intake during pregnancy	<1 time a month	284 (62.3)	1.75 ± 1.68	1.31 (0.64–2.35)	275 (61.5)	112 ± 122	79.6 (37.5–135)
	≥1 time a month	9 (1.97)	1.83 ± 1.94	0.89 (0.37–2.50)	9 (2.01)	98.5 ± 105	53.5 (23.7–107)
	Missing	163 (35.7)			163 (36.5)		
	*p value*	*0.83*			*0.71*		
	*Adjusted p value ^f^*	*0.85*			*0.77*		
Parity	0	176 (38.6)	2.00 ± 1.93	1.53 (0.76–2.62)	172 (38.5)	146 ± 147	98.6 (52.1–193)
	1–2	217 (47.6)	1.52 ± 1.30	1.25 (0.48–2.14)	214 (47.9)	103 ± 99.2	75.6 (25.8–137)
	≥3	48 (10.5)	1.68 ± 1.39	1.01 (0.53–3.03)	46 (10.3)	100 ± 86.6	78.7 (34.5–143)
	Missing	15 (3.29)			15 (3.36)		
	*p value*	*0.10*			***0.02***		
	*Adjusted p value ^g^*	***0.02***			***0.005***		

n: total number of participants having information for the parameter. p25: 25th percentile, p75: 75th percentile. *p* value was calculated with ANOVA and independent Student *t*–test analysis on ln–transformed data. Bold value indicates statistical significance (*p* < 0.050), # borderline significance (*p* < 0.080). ^a^ Adjusted *p* values were obtained from ANCOVA upon adjustment of BMI, n–3/n–6 PUFA ratio, education level, cotinine, alcohol intake during pregnancy and parity. ^b^ Adjusted *p* values were obtained from ANCOVA upon adjustment of age, n–3/n–6 PUFA ratio, education level, cotinine, alcohol intake during pregnancy and parity. ^c^ Adjusted *p* values were obtained from ANCOVA upon adjustment of age, BMI, n–3/n–6 PUFA ratio, cotinine, alcohol intake during pregnancy and parity. ^d^ Adjusted *p* values were obtained from ANCOVA upon adjustment of age, BMI, n–3/n–6 PUFA ratio, education level, alcohol intake during pregnancy and parity. ^e^ Adjusted *p* values were obtained from ANCOVA upon adjustment of age, BMI, n–3/n–6 PUFA ratio, education level, cotinine, alcohol intake during pregnancy and parity. ^f^ Adjusted *p* values were obtained from ANCOVA upon adjustment of age, BMI, n–3/n–6 PUFA ratio, education level, cotinine and parity. ^g^ Adjusted *p* values were obtained from ANCOVA upon adjustment of age, BMI, n–3/n–6 PUFA ratio, education level, cotinine, and alcohol intake during pregnancy.

**Table 3:** Maternal serum AhR–TEQ and fetal growth indices.

		AhR–TEQ (pg/mL)			AhR–TEQ (pg/g lipid)		
		n (%)	Mean ± SD	Median (p25–p75)	n	Mean ± SD	Median (p25–p75)
All		456 (100)	1.72 ± 1.60	1.32 (0.56–2.40)	447 (100)	119 ± 121	86.5 (35.1–160)
Birth weight (g)	Low (<2500)	16 (3.51)	1.77 ± 1.45	1.83 (0.24–3.09)	16 (3.58)	124 ± 107	118 (17.7–221)
	Normal (2500–4500)	423 (92.8)	1.72 ± 1.63	1.30 (0.56–2.37)	415 (92.8)	119 ± 123	85.7 (35.0–158)
	Macrosomia (≥4500)	17 (3.73)	1.59 ± 0.79	1.52 (1.08–2.37)	16 (3.58)	114 ± 66.0	113 (61.5–170)
	Missing	0 (0.00)			0 (0.00)		
	*p value*	*0.70*			*0.64*		
	Adjusted p value *	*0.19*			*0.07 ^#^*		
Birth length (cm)	<50	81 (17.8)	2.05 ± 1.95	1.67 (0.51–3.03)	80 (17.9)	141 ± 153	95.5 (31.1–213)
	≥50	375 (82.2)	1.65 ± 1.51	1.29 (0.56–2.29)	367 (82.1)	114 ± 112	85.2 (36.9–156)
	Missing	0 (0.00)					
	*p value*	*0.33*			*0.44*		
	Adjusted p value *	***0.05***			***0.05***		
Ponderal Index (g/cm^3^)	Normal (2.2–3.0)	407 (89.3)	1.75 ± 1.65	1.31 (0.57–2.47)	398 (89.0)	122 ± 125	85.8 (36.6–164)
	Abnormal (<2.2 or >3.0)	48 (10.5)	1.39 ± 1.00	1.45 (0.49–1.98)	48 (10.7)	93.8 ± 70.5	91.9 (26.4–131)
	Missing	1 (0.22)			1 (0.22)		
	*p value*	*0.16*			*0.20*		
	*Adjusted p value ^§^*	*0.17*			0.22		
Birth head circumference (cm)	<35	202 (44.3)	1.73 ± 1.72	1.31 (0.44–2.51)	196 (43.8)	116 ± 124	79.7 (28.7–159)
	≥35	253 (55.5)	1.71 ± 1.51	1.34 (0.63–2.37)	250 (55.9)	121 ± 119	89.7 (38.9–161)
	Missing	1 (0.22)			1 (0.22)		
	*p value*	*0.32*			*0.15*		
	Adjusted p value *	*0.26*			*0.17*		
Gestational age at birth (weeks)	Preterm birth (<37)	24 (5.26)	1.49 ± 1.83	0.89 (0.22–2.12)	24 (5.37)	93.8 ± 120	44.1 (7.36–451)
	Term birth (≥37)	417 (91.5)	1.75 ± 1.60	1.38 (0.59–2.44)	408 (91.3)	122 ± 122	89.1 (37.4–163)
	Missing	15 (5.86)			15 (3.36)		
	*p value*	*0.08 ^#^*			***0.05***		
	Adjusted p value *	*0.16*			*0.11*		
Gender	Male	241 (52.9)	1.70 ± 1.68	1.24 (0.54–2.37)	236 (52.8)	119 ± 123	85.1 (37.1–159)
	Female	214 (46.9)	1.73 ± 1.51	1.42 (0.56–2.42)	210 (47.0)	119 ± 118	88.8 (33.9–160)
	Missing	1 (0.22)			1 (0.22)		
	*p value*	*0.89*			*0.85*		
	Adjusted p value *	*0.58*			*0.62*		

n: total number of participants having information for the parameter. p25: 25th percentile, p75: 75th percentile. *p* value was calculated with ANOVA and independent Student *t*-test analysis on ln-transformed data. Bold value indicates statistical significance (*p* < 0.050), # borderline significance (*p* < 0.080). * Adjusted *p* values were obtained from ANCOVA upon adjustment of age, BMI, n-3/n-6 PUFA ratio, cotinine, alcohol intake during pregnancy, education level and parity. *^§^* Adjusted *p* values were obtained from ANCOVA upon adjustment of age, BMI, plasma cotinine, n-3/n-6 PUFA ratio, alcohol intake during pregnancy, parity, education level and gestational age.

**Table 4:** The association of serum lipPOPs, principle component and AhR–TEQ.

	AhR–TEQ (pg/mL)								
	Crude			Adjusted ^a^			Adjusted ^b^		
	n	β (95% CI)	*p*	n	β (95% CI)	*p*	n	β (95% CI)	*p*
PCB105 (µg/L)	465	1.65 (−3.56; 6.85)	0.53	465	1.54 (–3.94; 7.01)	0.58	258	6.00 (–4.24; 16.2)	0.25
PCB118 (µg/L)	465	0.16 (–0.73; 1.04)	0.73	465	0.13 (–0.80; 1.06)	0.79	258	0.96 (–1.17; 3.09)	0.38
PCB156 (µg/L)	465	2.30 (–0.55; 5.15)	0.11	465	2.39 (–0.61; 5.39)	0.12	258	6.59 (1.22; 12.0)	**0.02**
ΣDL–PCB (µg/L)	465	0.20 (–0.41; 0.83)	0.51	465	0.20 (–0.45; 0.86)	0.55	258	1.04 (–0.41; 2.49)	0.16
ΣPCB (µg/L)	465	0.01 (–0.03; 0.04)	0.72	465	0.01 (–0.03; 0.04)	0.77	258	0.09 (–0.02; 0.20)	0.11
ΣDL–PCB / ΣPCB	465	5.47 (–2.61; 13.6)	0.18	465	5.44 (–2.65; 13.5)	0.19	258	9.93 (–2.42; 22.3)	0.11
HCB (µg/L)	465	0.16 (–0.50; 0.81)	0.64	465	0.14 (–0.56; 0.84)	0.70	258	0.38 (–0.74; 1.50)	0.50
β–HCH (µg/L)	464	0.64 (–2.19; 3.47)	0.66	464	0.59 (–2.41; 3.59)	0.70	258	2.77 (–3.16; 8.70)	0.36
p,p’–DDE (µg/L)	465	0.00 (–0.03; 0.03)	0.98	465	–0.001 (–0.03; 0.03)	0.94	258	0.09 (–0.06; 0.24)	0.24
Oxychlordane (µg/L)	465	0.04 (–0.20; 0.28)	0.74	465	0.03 (–0.21; 0.28)	0.79	258	0.65 (–0.08; 1.37)	0.08 #
Transnonachlor (µg/L)	465	0.03 (–0.11; 0.16)	0.72	465	0.02 (–0.12; 0.16)	0.77	258	0.33 (–0.12; 0.78)	0.15
Mirex (µg/L)	465	0.45 (–1.25; 2.15)	0.60	465	0.42 (–1.34; 2.17)	0.64	258	3.91 (–0.89; 8.71)	0.11
ΣOCP (µg/L)	464	0.001 (–0.02; 0.02)	0.92	464	0.00 (–0.02; 0.02)	0.97	258	0.06 (–0.03; 0.14)	0.20
ΣLegacyPOP (µg/L)	464	0.001 (–0.01; 0.01)	0.85	463	0.001 (–0.01; 0.01)	0.89	257	0.04 (–0.01; 0.09)	0.15
ΣLipPOPs (µg/L)	464	0.001 (–0.01; 0.01)	0.84	463	0.001 (–0.01; 0.01)	0.66	257	0.04 (–0.01; 0.09)	0.20
**PCA component**									
PC–1 (lipPOP, ug/L)	457	0.04 (–0.10; 0.17)	0.60	253	0.25 (–0.16; 0.66)	0.24	253	0.31 (–0.13; 0.76)	0.17
PC–2 (Se and Hg, ug/L)	457	–0.03 (–0.17; 0.11)	0.63	253	0.07 (–0.13; 0.26)	0.48	253	0.08 (–0.12; 0.27)	0.45

^a^ adjusted for serum lipid; ^b^ adjusted for maternal age (year), pre–pregnancy BMI (kg/m^2^), plasma cotinine, n–3/n–6 PUFA ratio, alcohol intake during pregnancy, parity, serum lipid; Bold value indicates statistical significance (*p* ≤ 0.050), # indicate borderline significance (*p* ≤ 0.080). ΣDL–PCB: summation of dioxin–like PCB congener (PCB105, 118, 156); ΣPCB: summation of 14 PCB congeners; ΣDL–PCB/ΣPCB: ratio of dioxin–like PCB to sum of 14 PCBs; ΣOCP: summation of 11 OCP. ΣLegacy POPs included 14 PCBs and 11 OCPs, ΣLipPOPs: lipophilic POPs including 14 PCBs, 11 OCPs, 9 PBDEs and PBB153. PCA: principle component analysis; PC: principle component.

**Table 5:** Correlation of maternal characteristics with fetal growth indices and serum AhR–TEQ.

	Birth Weight (BW) (gram)			Birth Length (BL) (cm)			Ponderal Index (PI) (g/cm^3^)			Head Circumference (HC) (cm)			Gestational Age at Birth (GA) (Week)			AhR–TEQ (pg/mL Serum)			AhR–TEQ (pg/g Serum Lipid)		
	n	r_s_	*p*	n	r_s_	*p*	n	r_s_	*p*	n	r_s_	*p*	n	r_s_	*p*	n	r_s_	*p*	n	r_s_	p
Age (years)	456	0.05	0.25	456	0.06	0.20	456	0.02	0.66	455	0.08	0.11	441	0.03	0.49	456	0.04	0.40	447	0.02	0.76
Pre–pregnancy BMI (kg/m^2^)	442	0.15	**0.002**	442	0.11	**0.03**	442	0.11	**0.02**	441	0.12	**0.01**	428	0.04	0.38	442	–0.11	**0.02**	433	–0.11	**0.02**
Plasma cotinine (ng/mL)	444	–0.25	**<0.0001**	444	–0.26	**0.0001**	444	–0.03	0.50	443	–0.24	**<0.0001**	432	–0.15	**0.002**	444	0.05	0.27	435	0.04	0.47
Smoking history	455	–0.18	**<0.0001**	455	–0.24	**<0.0001**	455	0.03	0.56	454	–0.16	**<0.0001**	440	–0.12	**0.01**	455	0.04	0.44	446	0.05	0.26
n–3/n–6 PUFA ratio	455	0.04	0.38	455	0.03	0.58	455	–0.01	0.84	454	–0.04	0.43	440	0.09	**0.05**	455	0.06	0.20	446	0.10	**0.04**
Alcohol intake																					
before pregnancy	422	–0.03	0.57	422	–0.02	0.64	422	–0.04	0.46	421	–0.03	0.53	407	0.03	0.49	422	0.003	0.95	413	–0.02	0.72
during pregnancy	286	–0.08	0.20	286	–0.09	0.12	286	–0.01	0.85	285	–0.13	**0.03**	273	–0.04	0.49	286	–0.05	0.40	278	–0.06	0.30
Parity	441	0.02	0.73	441	–0.02	0.73	441	0.07	0.17	441	0.04	0.46	427	–0.04	0.43	441	–0.07	0.16	432	–0.11	**0.02**
Maternal education	455	0.10	**0.04**	455	0.10	**0.03**	455	0.02	0.61	454	0.14	**0.002**	440	0.07	0.17	455	0.04	0.40	446	0.06	0.40
Gestational age (GA) (week)	457	0.51	**<0.0001**	441	0.46	**<0.0001**	441	0.19	**<0.0001**	440	0.41	**<0.0001**	–	–	–	441	–0.05	0.29	432	0.001	0.99

r_s_: Spearman correlation coefficient; Bold value indicates statistical significance (*p* < 0.05). For smoking history and alcohol intake categories please consult Table 2.

## Data Availability

The data are not publicly available due to ethical considerations and privacy restrictions.

## Supplementary Materials

The following are available online at https://www.mdpi.com/article/10.3390/toxics10010026/s1, Figure S1: the association of quartiles of AhR-TEQ (pg/g) and fetal growth indices. Table S1: levels of lipophilic POPs and dioxin-like activity in maternal serum and fetal growth indices stratified by infant gender; Table S2: the detectable percentage of contaminants in ACCEPT Inuit pregnant women; Table S3: rotated factor loading of two components identified by principal component analysis of the ACCEPT Greenlandic pregnant women; Table S4: the associations of maternal serum AhR-TEQ (pg/g lipid) and fetal growth indices; Table S5: the association of maternal serum (AhR-TEQ, pg/g lipid) and fetal growth indices stratified on gender in the ACCEPT cohort; Table S6: the association of maternal serum AhR-TEQ (pg/g lipid) and fetal growth indices stratified on smoking history of the ACCEPT cohort; Table S7: the association of serum AhR-TEQ (pg/g lipid) and fetal growth indices stratified by gestation age in ACCEPT cohort.

## Abbreviations

ACCEPT	Adapting to Climate Change and Environmental Pollution and Dietary Transition
AhR	Aryl hydrocarbon receptor
AR	Androgen receptor
BMI	Body mass index
BL	Birth length
BW	Birth weight
ß-HCH	β-hexachlorocyclohexane
DL-PCB	Dioxin-like- polychlorinated biphenyl
DLCs	Dioxin-like compounds
DMSO	Dimethyl sulfoxide
EC_50_	Half maximum effect concentration
EDCs	Endocrine disrupting chemicals
ER	Estrogen receptor
GA	Gestational age at birth
HC	Head circumference
HCB	Hexachlorobenzene
Hg	Mercury
lipPOPs	Lipophilic persistent organic pollutants
LOD	Limit of detection
n-3	Omega-3 fatty acids
n-6	Omega-6 fatty acids
OCP	Organochlorine pesticide
PAHs	Polycyclic aromatic hydrocarbons
PBB	Polybrominated biphenyl
PBDE	Polybrominated diphenyl ethers
PC	Principal component
PCA	Principal component analysis
PI	Ponderal index
PCB	Polychlorinated biphenyl
PCDDs	Polychlorinated dibenzo-p-dioxins
PCDFs	Polychlorinated dibenzofurans
POP	Persistent organic pollutant
p,p′-DDE	Dichlorodiphenyldichloroethylene
p,p′-DDT	Dichlorodiphenyltrichloroethane
PUFA	Polyunsaturated fatty acid
RLU	Relative light unit
SC	Solvent control
Se	Selenium
SPE	Solid phase extraction
TCDD	2,3,7,8-tetrachlorodibenzo-p-dioxin
TEF	Toxic Equivalency Factor
TEQ	TCDD toxic equivalent
TR	Thyroid hormone receptor
