# Supplementary material for: Dioxin-like Activity in Pregnant Women and Indices of Fetal Growth: The ACCEPT Birth Cohort
Source: Toxics. 2022 Jan 8;10(1):26. doi: 10.3390/toxics10010026 (PMC8781564; doi:10.3390/toxics10010026)
Supplement: Supplementary file 1 [file toxics-10-00026-s001.zip › toxics-1501844- final supplementray.pdf]

toxics

Supplementary Materials: Dioxin-like Activity in Pregnant Women and Indices of Fetal Growth: The ACCEPT Birth Cohort

Manhai Long, Maria Wielsøe and Eva Cecilie Bonefeld-Jørgensen

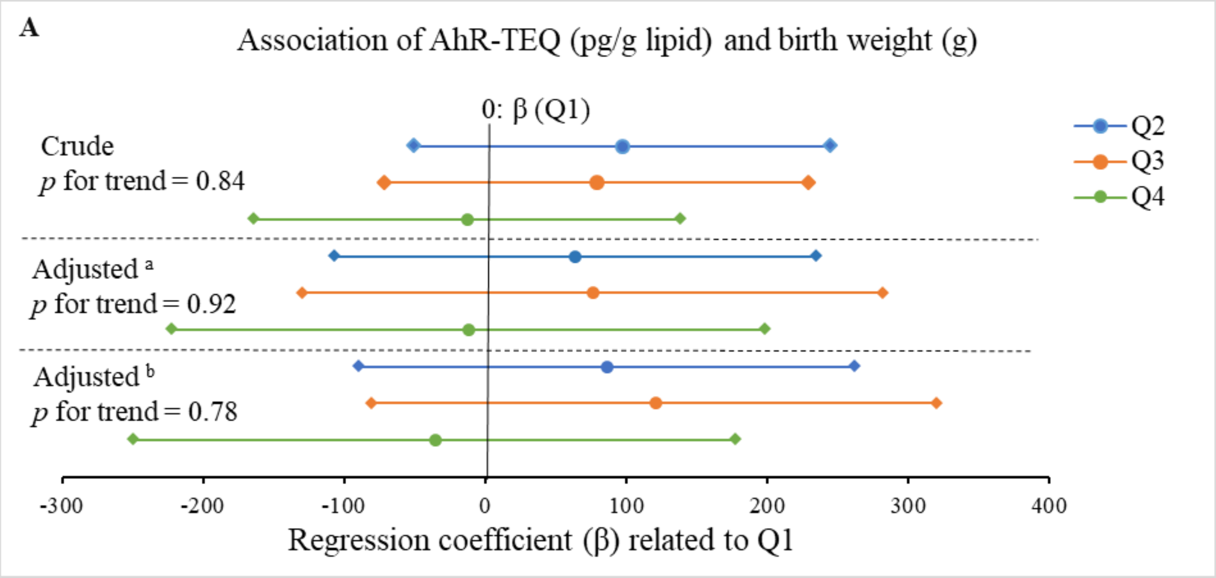

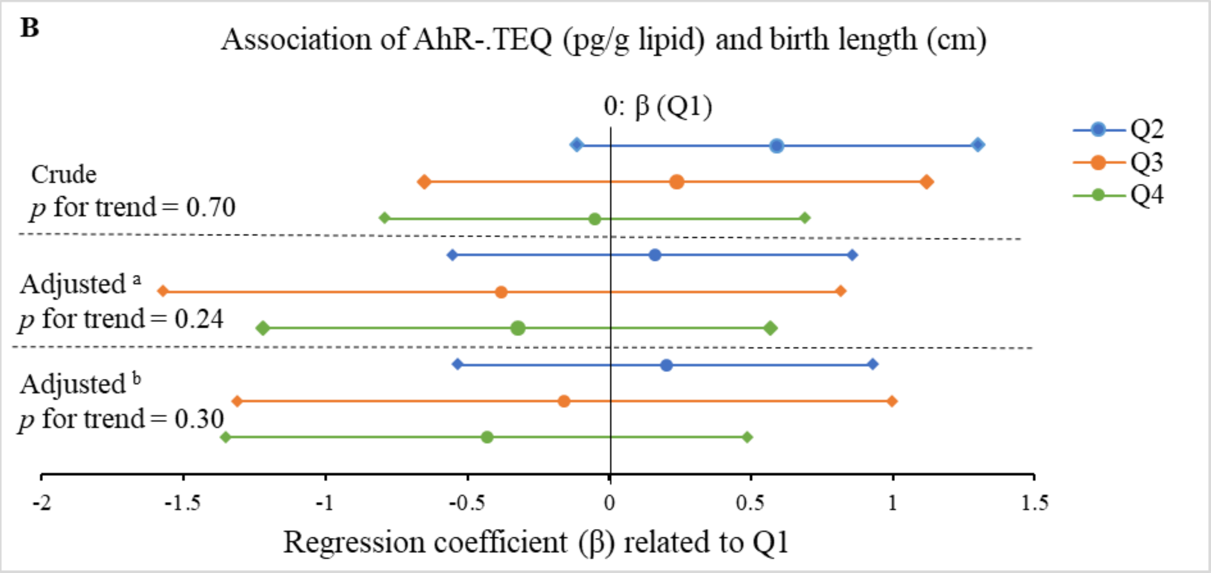

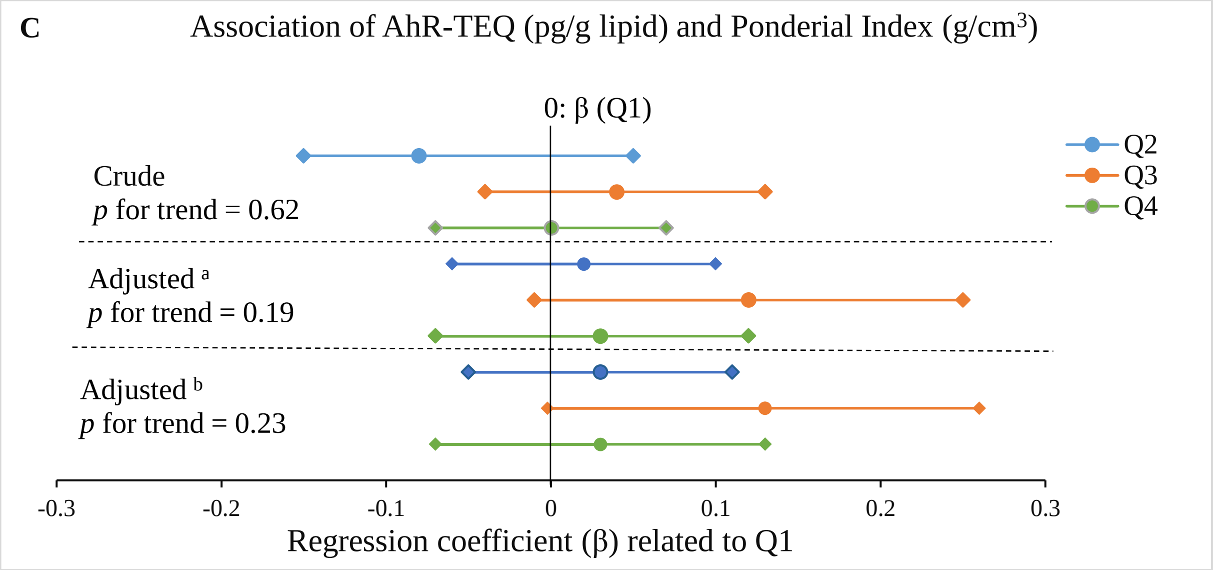
C
Association of AhR-TEQ (pg/g lipid) and Ponderial Index (g/cm3)
0: β (Q1)
Q2
Q3
Q4
Crude
p for trend = 0.62
Adjusted a
p for trend = 0.19
Adjusted b
p for trend = 0.23
-0.3 -0.2 -0.1 0 0.1 0.2 0.3
Regression coefficient (β) related to Q1

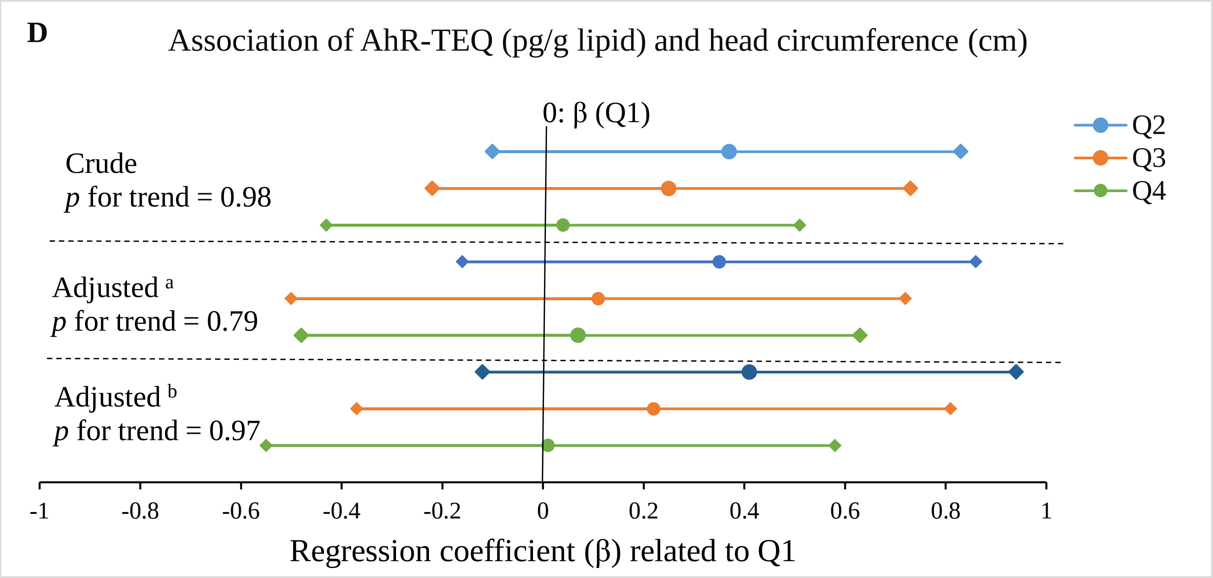
D
Association of AhR-TEQ (pg/g lipid) and head circumference (cm)
0: β (Q1)
Q2
Q3
Q4
Crude
p for trend = 0.98
Adjusted a
p for trend = 0.79
Adjusted b
p for trend = 0.97
-1 -0.8 -0.6 -0.4 -0.2 0 0.2 0.4 0.6 0.8 1
Regression coefficient (β) related to Q1

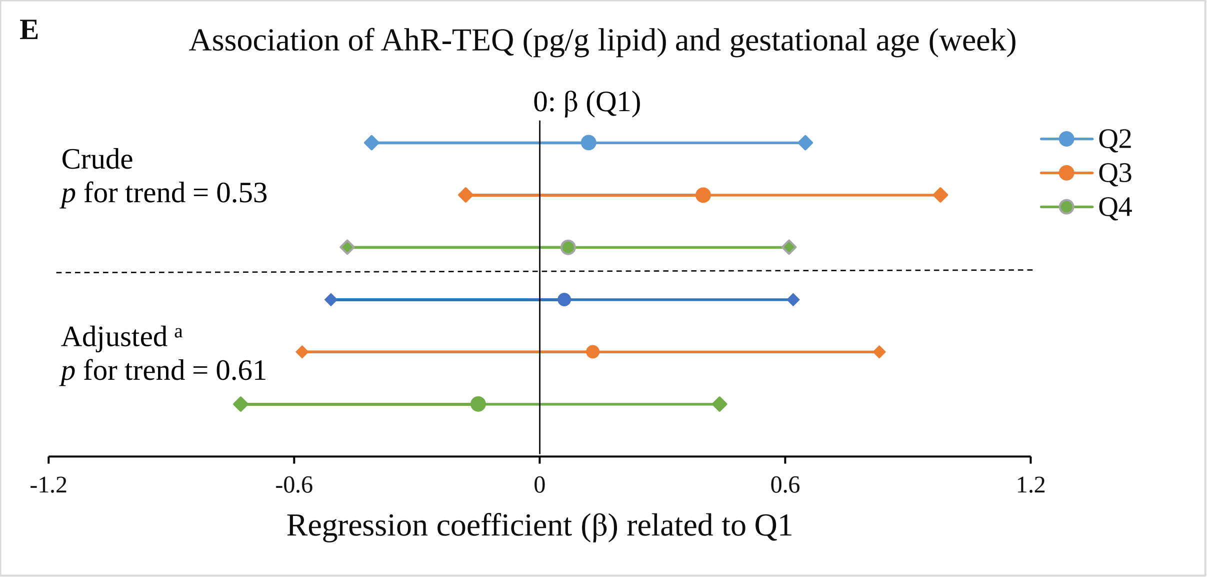
E
Association of AhR-TEQ (pg/g lipid) and gestational age (week)
0: β (Q1)
Q2
Q3
Q4
Crude
p for trend = 0.53
Adjusted a
p for trend = 0.61
-1.2 -0.6 0 0.6 1.2
Regression coefficient (β) related to Q1

Figure S1. The association of quartiles of AhR-TEQ (pg/g) and foetal growth indices. **A**) Association of AhR-TEQ (pg/g lipid) and birth weight (g); **B**) Association of AhR-TEQ and birth length (cm): **C**) Association of AhR-TEQ (pg/g lipid) and Ponderal Index (g/cm^3); **D**) Association of AhR-TEQ (pg/g lipid) and head circumference (cm); **E**) Association of AhR-TEQ (pg/g lipid) and gestational age (week). Adjusted [a]: adjusted for maternal age, pre-pregnancy BMI, alcohol consumption during pregnancy and parity. Adjusted [b]: adjusted for maternal age, pre-pregnancy BMI, alcohol consumption during pregnancy, parity and gestational age. Round circle: estimate (β). Diamond circle: end of 95% CI.

Table S1. Levels of lipophilic POPs and dioxin like activity in maternal serum and fetal growth indices stratified by infant gender[¤].

		**Male**	**Female**	***p***	**All**
**POPs (µg/kg serum lipid)**	n	237	211		449
PCB105	Mean ± SD	2.09 ± 2.23	2.72 ± 4.21		2.39 ± 3.33
	Median (p25–p75)	1.10 (1.00–2.40)	1.80 (1.00–2.98)	0.38	1.60 (1.00–2.60)
PCB118	Mean ± SD	11.8 ± 11.9	15.0 ± 25.9		13.3 ± 19.5
	Median (p25–p75)	8.80 (5.45–13.0)	9.10 (5.23–15.0)	0.49	9.00 (5.40–14.0)
PCB156	Mean ± SD	4.33 ± 4.10	5.05 ± 7.91		4.65 ± 6.19
	Median (p25–p75)	3.10 (1.95–5.30)	3.30 (2.00–5.48)	0.97	3.20 (2.00–5.40)
∑DL-PCB	Mean ± SD	18.2 ± 17.0	22.8 ± 36.5		20.3 ± 27.5
	Median (p25–p75)	13.8 (8.63–20.1)	13.6 (8.30–24.3)	0.55	14.1 (8.40–21.9)
∑PCB	Mean ± SD	252 ± 276	336 ± 738		289 ± 537
	Median (p25–p75)	183 (115–272)	174 (119–283)	0.48	181 (117–279)
∑DL-PCB/∑PCB (%)	Mean ± SD	0.08 ± 0.02	0.08 ± 0.02		0.08 ± 0.02
	Median (p25–p75)	7.38 (6.30–8.86)	7.61 (6.39–8.87)	0.47	7.42 (6.35–8.85)
∑OCP	Mean ± SD	336 ± 395	473 ± 1222		397 ± 873
	Median (p25–p75)	226 (147–386)	233 (140–425)	0.12	233 (143–391)
∑Legacy POPs	Mean ± SD	588 ± 664	809 ± 1950		687 ± 1401
	Median (p25–p75)	417 (268–639)	418 (268–690)	0.44	422 (270–674)
∑Lip POPs	Mean ± SD	606 ± 665	827 ± 1952		704 ± 1403
	Median (p25–p75)	433(279–654)	432 (284–712)	0.44	436 (281–684)
**Dioxin like activity**					
	n	241	214		456
AhR-TEQ (pg/ml serum)	Mean ± SD	1.70 ± 1.68	1.73 ± 1.51	0.89	1.72 ± 1.60
	Median (p25–p75)	1.24 (0.54–2.37)	1.42 (0.56–2.42)		1.32 (0.56–2.40)
	n	236	210		447
AhR-TEQ (pg/g serum lipid)	Mean ± SD	119 ± 123	119 ± 118	0.85	119 ± 121
	Median (p25–p75)	85.1 (37.1–159)	88.8 (33.9–160)		86.5 (35.0–160)
	n	237	211		449
**Serum lipid (g/l serum)**	Mean ± SD	7.33 ± 1.77	7.64 ± 1.86	0.07[#]	7.46 ± 1.80
	Median (p25–p75)	7.10 (5.95–8.55)	7.30 (6.40–8.88)		7.2 (6.10–8.70)
**Foetal growth indices**					
Birth weight (g)	n	241	214		456
	Mean ± SD	3677 ± 570	3467 ± 562	**0.001**	3578 ± 545
	Median (p25–p75)	3690 (3368–4010)	3458 (3201–3798)		3618 (3275–3930)

Birth length (cm)	n	241	214		456
	Mean ± SD	51.9 ± 2.51	50.7 ± 3.22	**< 0.0001**	51.3 ± 2.92
	Median (p25–p75)	52.0 (51.0–53.0)	51.0 (50.0–52.0)		51.0 (50.0–53.0)
Ponderal Index (g/cm^3)	n	241	214		456
	Mean ± SD	2.62 ± 0.25	2.66 ± 0.32	0.24	2.64 ± 0.28
	Median (p25–p75)	2.60 (2.48–2.75)	2.64 (1.97–5.68)		2.62 (2.49–2.77)
Head circumference (cm)	n	241	213		455
	Mean ± SD	34.9 ± 1.55	34.3 ± 1.85	**< 0.0001**	34.6 ± 1.73
	Median (p25–p75)	35.0 (34.0–36.0)	34.0 (33.5–35.0)		35.0 (34.0–36.0)
Gestational age at birth (week)	n	232	209		441
	Mean ± SD	39.2 ± 1.65	39.0 ± 2.15	0.25	39.1 ± 1.90
	Median (p25–p75)	39.0 (38.0–40.0)	39.0 (38.0–40.0)		39.0 (38.0–40.0)

¤ One subject had no gender information. n: total number of participants having information for the parameter. p value was calculated with independent student t-test analysis on ln-transformed data. DL-PCB (dioxin-like PCB) included PCB105, PCB118 and PCB156; ΣLegacy POPs included 14 PCBs and 11 OCPs, ΣLip POPs: lipophilic POPs including 14 PCBs, 11 OCPs and 10 PBDEs. Bold value indicates statistical significance ($p < 0.050$), # borderline significance ($p < 0.080$). p25: 25th percentile. p75: 75th percentile.

Table S2. The detectable percentage of contaminants in ACCEPT Inuit pregnant women.

Contaminant	% over LOD	Contaminant	% over LOD
**PCBs (n = 464)**		**PBDEs and PBB (n = 464)**	
PCB 138	100	PBDE 47	4.44
PCB 153	100	PBDE 153	3.09
PCB 180	100	PBDE 99	2.94
PCB 170	99.0	PBDE 100	1.03
PCB 118	98.6	PBB 153	0.69
PCB 187	98.3	PBDE 15	0
PCB 183	82.1	PBDE 17	0
PCB 156	80.4	PBDE 25	0
PCB 99	72.5	PBDE 28	0
PCB 105	44.4	PBDE 33	0
PCB 128	15.6		
PCB 101	7.01		
PCB 28	6.30		
PCB 52	0		
**OCPs (n = 464)**		**Metals (n = 468)**	
Hexachlorobenzene (HCB)	99.7	Se	100
p,p'-DDE	99.3	Plasma Se	99.8
Oxychlordane	98.3	Hg	78.6
trans-Nonachlor	98.1		
cis-Nonachlor	94.4		
ß-HCH	91.0		
Mirex	70.6		
p,p'-DDT	15.7		
α-Chlordane	1.03		
γ-Chlordane	0		
Aldrin	0		

LOD: limit of detection, PCB: Polychlorinated biphenyl, OCPs: Organochlorine pesticides, p,p'-DDE: Dichlorodiphenyldichloroethylene, β-HCH: β-hexachlorocyclohexane, p,p'-DDT: Dichlorodiphenyltrichloroethane, PBDEs: Polybrominated diphenyl ethers, PBB: Polybrominated biphenyl.

Table S3. Rotated factor loading of two components identified by principal component analysis (PCA) of the ACCEPT Greenlandic pregnant women.

Interpreted variable component	Variable name	Factor loading [a]	Explained variance	Explained variance cumulative
**PC-1 (lipPOP component, μg/L)**	PCB153	0.990	75.53%	75.53%
	PCB138	0.982		
	PCB187	0.980		
	trans-nonachlor	0.980		
	Mirex	0.978		
	oxychlordane	0.982		
	PCB183	0.971		
	p,p′-DDE	0.957		
	PCB180	0.969		
	PCB99	0.970		
	PCB118	0.952		
	PCB170	0.934		
	β-HCH	0.867		
	cis-nonachlor	0.903		
	PCB105	0.885		
	PCB156	0.856		
	HCB	0.801		
**PC-2 (fatty metal component, μg/L)**	Se	0.874	11.33%	86.85%
	Hg	0.817		
	Plasma Se	0.544		

[a] Factor loadings are the correlation coefficients between the original variables (contaminants concentrations) and the extracted components. Variables are sorted by the size of the factor loading. Variables with factor loading below 0.40 are not listed. PC: principle component. The PCA was performed for the contaminants detected in above 40% samples.

Table S4. The associations of maternal serum AhR-TEQ (pg/g lipid) and foetal growth indices.

	Crude			Adjusted [a]			Adjusted [b]		
	n	β (95% CI)	*p*	n	β (95% CI)	*p*	n	β (95% CI)	*p*
Birth weight (g)	447	−0.30 (−0.74; 0.14)	0.18	256	−0.21 (−0.79; 0.36)	0.47	246	−0.11 (−0.54; 0.31)	0.60
Birth length (cm)	447	−0.001 (−0.003; 0.001)	0.35	256	−0.001 (−0.004; 0.001)	0.32	246	−0.001 (−0.003; 0.001)	0.43
Ponderal Index (g/cm^3)	447	$-5.8e^{-5}$ ($-3e^{-4}$; $2e^{-4}$)	0.60	256	$1.2e^{-5}$($-3e^{-4}$; $3e^{-4}$)	0.94	246	$4.5e^{-7}$ ($-3e^{-4}$; $3e^{-4}$)	0.99
Head circumference (cm)	446	$-5.0e^{-4}$ (−0.002; 0.001)	0.51	256	$-4.0e^{-4}$ (−0.002; 0.001)	0.66	231	$-2.0e^{-4}$ (−0.001; 0.001)	0.81
Gestational age at birth (week)	432	$-8.9e^{-5}$ (−0.002; 0.001)	0.91	246	$4.0e^{-4}$ (−0.002; 0.001)	0.81	–	–	

β (95% CI): regression coefficient (95% confidential interval). [a]adjusted for maternal age (year), pre-pregnancy BMI (kg/m^2), plasma cotinine, education level, alcohol intake during pregnancy and parity; [b]adjusted for maternal age (year), pre-pregnancy BMI (kg/m^2), plasma cotinine, education level, alcohol intake during pregnancy, parity and gestational age at birth.

Table S5. The association of maternal serum (AhR-TEQ, pg/g lipid) and foetal growth indices stratified on gender in the ACCEPT cohort.

		Crude			Adjusted[a]			Adjusted[b]		
	AhR-TEQ (pg/g lipid)	n	β (95% CI)	*p*	n	β (95% CI)	*p*	n	β (95% CI)	*p*
**Male infant**										
Birth weight (BW)(g)	Continuous	236	−0.52 (−1.10; 0.07)	0.08#	127	−0.23 (−1.02; 0.57)	0.57	119	−0.13 (−0.72; 0.46)	0.67
	Q1 (0.224–34.20)	56	0 (reference)		33	0 (reference)		30	0 (reference)	
	Q2 (34.21–86.20)	65	118 (−63.4; 299)	0.20	40	112 (−123; 350)	0.35	38	120 (−133; 372)	0.35
	Q3 (86.21–159.8)	57	72.9 (−115; 261)	0.45	26	169 (−150; 488)	0.30	25	275 (−7.15; 557)	0.06#
	Q4 (> 159.8)	58	−62.5 (−266; 141)	0.55	28	21.0 (−294; 336)	0.90	26	−20.8 (−349; 308)	0.90
	p for trend		*0.47*			*0.81*			*0.88*	
Birth length (BL)(cm)	Continuous	236	−0.002 (−0.005; $1e^{-4}$)	0.06#	127	−0.002 (−0.006; 0.001)	0.18	119	−0.002 (−0.005; 0.001)	0.18
	Q1 (0.224–34.20)	56	0 (reference)		33	0 (reference)		30	0 (reference)	
	Q2 (34.21–86.20)	65	0.46 (−0.26; 1.18)	0.21	40	0.13 (−0.75; 1.00)	0.78	38	0.05 (−0.89; 0.98)	0.92
	Q3 (86.21–159.8)	57	−0.02 (−0.94; 0.90)	0.96	26	−0.46 (−2.05; 1.12)	0.57	25	0.05 (−1.21; 1.30)	0.94
	Q4 (> 159.8)	58	−0.58 (−1.46; 0.30)	0.20	28	−0.68 (−1.97; 0.61)	0.30	26	−0.92 (−2.27; 0.43)	0.18
	p for trend		*0.12*			*0.17*			*0.08#*	
Ponderal Index (PI)(g/cm³)	Continuous	236	$-6.3e^{-5}$ ($-3e^{-4}$; $2e^{-4}$)	0.63	127	$1.2e^{-4}$ ($-2e^{-4}$; $5e^{-4}$)	0.49	119	$1.4e^{-4}$ ($-2e^{-4}$; $4e^{-4}$)	0.38
	Q1 (0.224–34.20)	56	0 (reference)		33	0 (reference)		30	0 (reference)	
	Q2 (34.21–86.20)	65	0.003 (−0.09; 0.09)	0.95	40	0.06 (−0.06; 0.17)	0.31	38	0.10 (−0.03; 0.23)	0.20
	Q3 (86.21–159.8)	57	0.05 (−0.05; 0.14)	0.34	26	0.18 (0.04; 0.31)	**0.01**	25	0.19 (0.05; 0.33)	**0.01**
	Q4 (> 159.8)	58	0.02 (−0.08; 0.11)	0.72	28	0.09 (−0.03; 0.21)	0.14	26	0.02 (−0.11; 0.16)	0.13
	p for trend		*0.51*			***0.03***			***0.02***	
Head circumference (HC)(cm)	Continuous	236	−0.001 (−0.003; $3e^{-4}$)	0.10	127	−0.001 (−0.003; 0.001)	0.33	119	−0.001 (−0.002; 0.001)	0.30
	Q1 (0.224–34.20)	56	0 (reference)		33	0 (reference)		30	0 (reference)	
	Q2 (34.21–86.20)	65	0.30 (−0.20; 0.80)	0.25	40	0.45 (−0.21; 1.11)	0.18	38	0.45 (−0.25; 1.16)	0.21
	Q3 (86.21–159.8)	57	0.08 (−0.44; 0.59)	0.77	26	0.25 (−0.61; 1.11)	0.57	25	0.52 (−0.20; 1.24)	0.16
	Q4 (> 159.8)	58	−0.24 (−0.84; 0.35)	0.42	28	−0.04 (−0.82; 0.73)	0.91	26	−0.17 (−0.98; 0.64)	0.68
	p for trend		*0.28*			*0.77*			*0.55*	
Gestational age at birth (GA) (week)	Continuous	227	$-4.0e^{-4}$ (−0.002; 0.001)	0.62	119	$-3.4e^{-4}$ (−0.003; 0.002)	0.76	–	–	–
	Q1 (0.224–34.20)	52	0 (reference)		30	0 (reference)		–	–	–
	Q2 (34.21–86.20)	63	−0.02 (−0.61; 0.58)	0.96	38	0.13(−0.73; 0.99)	0.76	–	–	–
	Q3 (86.21–159.8)	56	0.58 (−0.02; 1.18)	0.06#	25	0.89 (0.00; 1.79)	**0.05**	–	–	–
	Q4 (> 159.8)	56	−0.09 (−0.75; 0.57)	0.78	26	−0.10 (−0.99; 0.79)	0.83	–	–	–
	p for trend		*0.96*			*0.87*		–	–	–
**Female infant**										
Birth weight (g)	Continuous	210	−0.03 (−0.68; 0.63)	0.94	129	−0.34 (−1.22; 0.53)	0.44	127	−0.14 (−0.77; 0.49)	0.66
	Q1 (0.224–34.20)	56	0 (reference)		31	0 (reference)		31	0 (reference)	
	Q2 (34.21–86.20)	48	37.3 (-186; 267)	0.74	36	6.79 (−231; 245)	0.96	34	40.8 (−199; 280)	0.74
	Q3 (86.21–159.8)	55	87.4 (−142; 317)	0.46	39	40.2 (−227; 307)	0.77	39	40.2 (−227; 307)	0.77
	Q4 (> 159.8)	51	39.5 (−181; 261)	0.73	23	−61.0 (−319; 197)	0.64	23	−61.0 (−319; 197)	0.64
	p for trend		*0.61*			*0.75*			*0.76*	
Birth length (cm)	Continuous	210	0.001 (−0.003; 0.004)	0.70	129	−0.001 (−0.007; 0.004)	0.60	127	$-1.0e^{-4}$ (−0.004; 0.004)	0.95
	Q1 (0.224–34.20)	56	0 (reference)		31	0 (reference)		31	0 (reference)	
	Q2 (34.21–86.20)	48	0.52 (−0.64; 1.69)	0.38	36	0.16 (−0.87; 1.19)	0.76	34	0.27 (−0.77; 1.31)	0.62
	Q3 (86.21–159.8)	55	0.51 (−0.97; 2.00)	0.50	39	−0.06 (−1.74; 1.63)	0.95	39	−0.06 (−1.74; 1.63)	0.95
	Q4 (> 159.8)	51	0.54 (−0.62; 1.69)	0.36	23	0.007 (−1.14; 1.16)	0.99	23	0.007 (−1.14; 1.16)	0.99
	p for trend		*0.41*			*0.87*			*0.56*	
Ponderal Index (g/cm³)	Continuous	210	$-6.5e^{-5}$ ($-4e^{-4}$; $3e^{-4}$)	0.73	129	$3.1e^{-5}$ (−0.001; 0.001)	0.92	127	$-1.1e^{-4}$ (−0.001; $5e^{-4}$)	0.71

	Q1 (0.224–34.20)	56	0 (reference)		31	0 (reference)		31	0 (reference)	
	Q2 (34.21–86.20)	48	−0.03 (−0.11; 0.05)	0.47	36	−0.03 (−0.13; 0.08)	0.61	34	−0.02 (−0.13; 0.09)	0.76
	Q3 (86.21–159.8)	55	0.04 (−0.10; 0.18)	0.59	39	0.06 (−0.13; 0.25)	0.52	39	0.06 (−0.13; 0.25)	0.52
	Q4 (> 159.8)	51	−0.02 (−0.12; 0.07)	0.65	23	−0.04 (−0.19; 0.10)	0.55	23	−0.04 (−0.90; 0.10)	0.55
	p for trend		*0.99*			*0.98*			*0.74*	
Head circumference (cm)	Continuous	209	0.001 (−0.001; 0.003)	0.52	129	$-1.0e^{-4}$ (−0.003; 0.003)	0.94	127	$4.0e^{-4}$ (−0.002; 0.003)	0.72
	Q1 (0.224–34.20)	56	0 (reference)		31	0 (reference)		31	0 (reference)	
	Q2 (34.21–86.20)	47	0.32 (-0.46; 1.10)	0.43	36	0.24 (−0.53; 0.99)	0.55	34	0.32 (-0.45; 1.09)	0.42
	Q3 (86.21–159.8)	55	0.44 (−0.34; 1.22)	0.27	39	0.11 (−0.72; 0.94)	0.79	39	0.11 (−0.72; 0.94)	0.79
	Q4 (> 159.8)	51	0.35 (−0.36; 1.05)	0.34	23	0.18 (−0.59; 0.96)	0.65	23	0.18 (−0.60; 0.96)	0.65
	p for trend		*0.29*			*0.93*			*0.54*	
Gestational age at birth (week)	Continuous	205	$3.0e^{-4}$ (−0.002; 0.003)	0.80	127	−0.001 (−0.004; 0.002)	0.50	–	–	–
	Q1 (0.224–34.20)	56	0 (reference)		31	0 (reference)		–	–	–
	Q2 (34.21–86.20)	44	0.25 (−0.63; 1.12)	0.58	34	−0.009 (−0.73; 0.71)	0.98	–	–	–
	Q3 (86.21–159.8)	54	0.22 (−0.76; 1.19)	0.67	39	−0.40 (−1.37; 0.56)	0.41	–	–	–
	Q4 (> 159.8)	51	0.34 (−0.50; 1.18)	0.43	23	−0.18 (−0.93; 0.57)	0.63	–	–	–
	p for trend		*0.45*			*0.45*		–	–	–

β (95% CI): regression coefficient (95% confidential interval). [a]adjusted for maternal age (year), pre-pregnancy BMI (kg/m^2), plasma cotinine, educational level, alcohol intake during pregnancy, parity; [b]adjusted for maternal age (year), pre-pregnancy BMI (kg/m^2), plasma cotinine, education level, alcohol intake during pregnancy, parity, gestational age at birth. Bold value indicates statistical significance ($p \leq 0.050$), # borderline significance ($p \leq 0.080$).

Table S6. The association of maternal serum AhR-TEQ (pg/g lipid) and foetal growth indices stratified on smoking history of the ACCEPT cohort.

		Crude			Adjusted[a]			Adjusted[b]		
	AhR-TEQ (pg/g lipid)	n	β (95% CI)	*p*	n	β (95% CI)	*p*	n	β (95% CI)	*p*
**Never smokers**										
Birth weight (g)	Continuous	107	−0.21 (−1.10; 0.68)	0.64	81	0.19 (−0.84; 1.21)	0.72	76	0.25 (−0.63; 1.12)	0.57
	Q1 (0.224–34.20)	31	0 (reference)		25	0 (reference)		24	0 (reference)	
	Q2 (34.21–86.20)	29	28.1 (−213; 269)	0.82	24	39.4 (−200; 279)	0.75	21	68.1 (−176; 313)	0.59
	Q3 (86.21–159.8)	22	−61.7 (−415; 291)	0.73	19	−27.6 (−412; 357)	0.89	18	94.3 (−234; 422)	0.57
	Q4 (> 159.8)	25	−131 (−411; 149)	0.36	13	−57.9 (−399; 284)	0.74	13	−53.5 (−398; 291)	0.76
	p for trend		*0.33*			*0.66*			*0.98*	
Birth length (cm)	Continuous	107	−2.2e^{-4} (−0.005; 0.004)	0.92	81	3.9e^{-4} (−0.005; 0.005)	0.88	76	0.001 (−0.003; 0.005)	0.73
	Q1 (0.224–34.20)	31	0 (reference)		25	0 (reference)		24	0 (reference)	
	Q2 (34.21–86.20)	29	0.37 (−0.79; 1.53)	0.53	24	0.04 (−1.01; 1.09)	0.94	21	0.06 (−1.01; 1.13)	0.91
	Q3 (86.21–159.8)	22	−0.95 (−2.82; 0.92)	0.32	19	−1.47 (−3.43; 0.49)	0.14	18	−0.86 (−2.37; 0.65)	0.26
	Q4 (> 159.8)	25	−0.42 (−1.71; 0.86)	0.52	13	−0.46 (−1.95; 1.02)	0.54	13	−0.55 (−2.03; 0.94)	0.47
	p for trend		*0.30*			*0.28*			*0.39*	
Ponderal Index (g/cm^3)	Continuous	107	−1.5e^{-4} (−0.001; 2.1e^{-4})	0.41	81	−2.1e^{-5} (−3.9e^{-4}; 3e^{-4})	0.92	76	3.3e^{-5} (−3.6e^{-4}; 4.3e^{-4})	0.87
	Q1 (0.224–34.20)	31	0 (reference)		25	0 (reference)		24	0 (reference)	
	Q2 (34.21–86.20)	29	−0.04 (−0.14; 0.06)	0.46	24	0.02 (−0.10; 0.13)	0.79	21	0.04 (−0.08; 0.15)	0.53
	Q3 (86.21–159.8)	22	0.08 (−0.07; 0.24)	0.29	19	0.17 (0.02; 0.33)	**0.03**	18	0.19 (0.04; 0.35)	**0.02**
	Q4 (> 159.8)	25	−0.05 (−0.15; 0.06)	0.38	13	0.01 (−0.11; 0.13)	0.88	13	0.03 (−0.10; 0.15)	0.67
	p for trend		*0.91*			*0.39*			*0.27*	
Head circumference (cm)	Continuous	107	−0.001 (−0.003; 0.002)	0.61	81	2.0e^{-4} (−0.003; 0.003)	0.89	76	−3.6e^{-4} (−0.002; 0.003)	0.77
	Q1 (0.224–34.20)	31	0 (reference)		25	0 (reference)		24	0 (reference)	
	Q2 (34.21–86.20)	29	0.34 (−0.38; 1.06)	0.35	24	0.55 (−0.22; 1.31)	0.16	21	0.63 (−0.19; 1.44)	0.13
	Q3 (86.21–159.8)	22	−0.19 (−1.16; 0.78)	0.71	19	−0.08 (−1.16; 0.99)	0.88	18	0.30 (−0.57; 1.17)	0.50
	Q4 (> 159.8)	25	−0.35 (−1.09; 0.39)	0.35	13	−0.003 (−0.77; 0.77)	0.99	13	0.01 (−0.77; 0.80)	0.97
	p for trend		*0.26*			*0.77*			*0.95*	
Gestational age at birth (week)	Continuous	102	−0.001 (−0.003; 0.002)	0.67	76	3.1e^{-4} (−0.002; 0.003)	0.81	–	–	–
	Q1 (0.224–34.20)	30	0 (reference)		24	0 (reference)		–	–	–
	Q2 (34.21–86.20)	26	−0.03 (−0.89; 0.83)	0.94	21	−0.25 (−1.07; 0.57)	0.55	–	–	–
	Q3 (86.21–159.8)	21	0.27 (−0.60; 1.14)	0.54	18	0.25 (−0.60; 1.10)	0.56	–	–	–
	Q4 (> 159.8)	25	−0.30 (−1.15; 0.55)	0.49	13	−0.25 (−1.01; 0.51)	0.52	–	–	–
	p for trend		*0.65*			*0.77*				
**Previous smokers**										
Birth weight (g)	Continuous	172	−0.76 (−1.44; 0.09)	**0.03**	85	−1.43 (−2.41; −0.44)	**0.01**	83	−0.84 (−1.60; -0.08)	**0.03**
	Q1 (0.224–34.20)	45	0 (reference)		16	0 (reference)		16	0 (reference)	
	Q2 (34.21–86.20)	39	201 (−19.4; 422)	0.07#	23	120 (−165; 404)	0.41	22	114 (−178; 406)	0.44
	Q3 (86.21–159.8)	45	103 (−75.1; 281)	0.26	22	141 (−103; 385)	0.26	22	141 (−103; 385)	0.26
	Q4 (> 159.8)	43	−100 (−332; 132)	0.40	24	−155 (−464; 155)	0.33	23	−173 (−489; 143)	0.28
	p for trend		*0.30*			*0.34*			*0.28*	
Birth length (cm)	Continuous	172	-0.003 (-0.006; −7.2e^{-4})	**0.05**	85	−0.006 (−0.01; −0.002)	**0.01**	83	−0.004 (−0.008; −3.2e^{-4})	**0.05**
	Q1 (0.224–34.20)	45	0 (reference)		16	0 (reference)		16	0 (reference)	
	Q2 (34.21–86.20)	39	0.94 (0.02; 1.85)	**0.05**	23	0.47 (−0.77; 1.72)	0.46	22	0.44 (−0.82; 1.71)	0.49
	Q3 (86.21–159.8)	45	0.64 (−0.18; 1.47)	0.13	22	0.49 (−0.73; 1.71)	0.43	22	0.49 (−0.73; 1.71)	0.43
	Q4 (> 159.8)	43	−0.46 (−1.51; 0.59)	0.39	24	−0.75 (−2.24; 0.74)	0.32	23	−0.83 (−2.35; 0.68)	0.28
	p for trend		*0.33*			*0.32*			*0.32*	
Ponderal Index (g/m^3)	Continuous	172	−1.1e^{-4} (−4.3e^{-4}; 2.1e^{-4})	0.51	85	−1.4e^{-4} (−0.001; 3.4e^{-4})	0.57	83	0.003 (−0.01; 0.02)	0.60
	Q1 (0.224–34.20)	45	0 (reference)		16	0 (reference)		16	0 (reference)	

	Q2 (34.21–86.20)	39	−0.009 (−0.10; 0.08)	0.84	23	0.002 (−0.12; 0.12)	0.97	22	0.003 (−0.12; 0.13)	0.97
	Q3 (86.21–159.8)	45	−0.02 (−0.12; 0.08)	0.71	22	0.03 (−0.11; 0.17)	0.67	22	0.03 (−0.11; 0.17)	0.67
	Q4 (> 159.8)	43	−0.02 (−0.13; 0.09)	0.76	24	−0.01 (−0.15; 0.13)	0.91	23	−0.01 (−−0.16; 0.14)	0.90
	p for trend		*0.70*			*0.89*			*0.93*	
Head circumference (cm)	Continuous	172	−0.002 (−0.004; $3.2e^{-4}$)	0.10	85	−0.003 (−0.006; −0.001)	**0.01**	83	−0.003 (−0.005; $-2.5e^{-4}$)	**0.05**
	Q1 (0.224–34.20)	45	0 (reference)		16	0 (reference)		16	0 (reference)	
	Q2 (34.21–86.20)	39	0.40 (−0.23; 1.03)	0.21	23	−0.05 (−0.89; 0.79)	0.91	22	0.01 (−0.84; 0.87)	0.98
	Q3 (86.21–159.8)	45	0.33 (−0.16; 0.82)	0.18	22	0.15 (-0.55; 0.84)	0.68	22	0.15 (−0.55; 0.84)	0.68
	Q4 (> 159.8)	43	−0.28 (−0.96; 0.40)	0.42	24	−0.69 (−1.53; 0.15)	0.11	23	−0.70 (−1.56; 0.16)	0.11
	p for trend		*0.42*			*0.11*			*0.10*	
Gestational age at birth (week)	Continuous	169	−0.002 (−0.004; $1.8e^{-4}$)	0.07#	83	−0.003 (−0.006; $3.5e^{-4}$)	0.08#	–	–	–
	Q1 (0.224–34.20)	45	0 (reference)		16	0 (reference)		–	–	–
	Q2 (34.21–86.20)	38	0.06 (−0.59; 0.70)	0.86	22	0.13 (−0.89; 1.14)	0.81	–	–	–
	Q3 (86.21–159.8)	44	0.69 (0.10; 1.28)	**0.02**	22	0.44 (−0.54; 1.43)	0.38	–	–	–
	Q4 (> 159.8)	42	−0.34 (−1.05; 0.38)	0.36	23	−0.38 (−1.37; 0.62)	0.46	–	–	–
	p for trend		*0.77*			*0.73*				
**Current smokers during pregnancy**										
Birth weight (g)	Continuous	163	0.07 (−0.64; 0.78)	0.84	93	−0.15 (−1.08; 0.77)	0.75	89	−0.09 (−0.69; 0.51)	0.77
	Q1 (0.224–34.20)	37	0 (reference)		24	0 (reference)		22	0 (reference)	
	Q2 (34.21–86.20)	44	139 (−118; 395)	0.29	31	69.3 (−201; 340)	0.62	30	123 (−158; 403)	0.39
	Q3 (86.21–159.8)	41	182 (−96.3; 461)	0.20	24	89.0 (−269; 447)	0.63	24	133 (−231; 496)	0.47
	Q4 (> 159.8)	41	206 (−57.5; 469)	0.13	14	62.0 (−348; 473)	0.77	13	2.49 (−382; 387)	0.99
	p for trend		*0.12*			*0.37*			*0.10*	
Birth length (cm)	Continuous	163	$4.6e^{-4}$ (−0.004; 0.005)	0.83	93	−0.001 (−0.007; 0.004)	0.67	89	−0.001 (−0.005; 0.003)	0.70
	Q1 (0.224–34.20)	37	0 (reference)		24	0 (reference)		22	0 (reference)	
	Q2 (34.21–86.20)	44	0.89 (−0.54; 2.31)	0.22	31	0.37 (−0.72; 1.47)	0.50	30	0.60 (−0.53; 1.73)	0.30
	Q3 (86.21–159.8)	41	0.81 (-1.12; 2.74)	0.41	24	−0.08 (−2.59; 2.42)	0.95	24	0.13 (−2.39; 2.64)	0.92
	Q4 (> 159.8)	41	0.96 (−0.50; 2.41)	0.20	14	0.15 (−1.36; 1.65)	0.85	13	7.44e-15 (−1.42; 1.42)	1.00
	p for trend		*0.27*			*0.94*			*0.57*	
Ponderal Index (g/m³)	Continuous	163	$1.4e^{-5}$ ($-4e^{-4}$; $4e^{-4}$)	0.95	93	$2.31e^{-5}$ (−0.001; 0.001)	0.95	89	$-6.2e^{-6}$ (−0.001; 0.001)	0.99
	Q1 (0.224–34.20)	37	0 (reference)		24	0 (reference)		22	0 (reference)	
	Q2 (34.21–86.20)	44	$7.8e^{-5}$ (−0.11; 0.11)	0.99	31	−0.01 (−0.16; 0.15)	0.94	30	$3.65e^{-4}$ (−0.16; 0.16)	0.99
	Q3 (86.21–159.8)	41	0.08 (−0.11; 0.26)	0.42	24	0.12 (-0.17; 0.42)	0.41	24	0.13 (−0.17; 0.43)	0.41
	Q4 (> 159.8)	41	0.04 (−0.08; 0.16)	0.50	14	0.002 (−0.19; 0.20)	0.99	13	−0.01 (−0.22; 0.19)	0.89
	p for trend		*0.42*			*0.38*			*0.53*	
Head circumference (cm)	Continuous	162	0.001 (−0.002; 0.003)	0.49	93	$3.0e^{-4}$ (−0.003; 0.003)	0.83	89	$3.6e^{-4}$ (−0.002; 0.002)	0.72
	Q1 (0.224–34.20)	37	0 (reference)		24	0 (reference)		22	0 (reference)	
	Q2 (34.21–86.20)	43	0.62 (−0.33; 1.56)	0.20	31	0.51 (−0.27; 1.28)	0.20	30	0.65 (−0.15; 1.46)	0.11
	Q3 (86.21–159.8)	41	0.58 (−0.43; 1.58)	0.26	24	0.17 (−0.91; 1.25)	0.76	24	0.24 (−0.86; 1.34)	0.67
	Q4 (> 159.8)	41	0.76 (−0.17; 1.69)	0.11	14	0.58 (−0.51; 1.66)	0.30	13	0.40 (−0.65; 1.45)	0.45
	p for trend		*0.11*			*0.28*			*0.11*	
Gestational age at birth (week)	Continuous	156	0.002 (−0.001; 0.005)	0.19	89	$-2.3e^{-4}$ (−0.004; 0.003)	0.90	–	–	–
	Q1 (0.224–34.20)	34	0 (reference)		22	0 (reference)		–	–	–
	Q2 (34.21–86.20)	42	0.56 (−0.66; 1.78)	0.37	30	0.17 (−0.86; 1.19)	0.75	–	–	–
	Q3 (86.21–159.8)	41	0.41 (−0.96; 178)	0.56	24	−0.21 (−1.68; 1.27)	0.78	–	–	–
	Q4 (> 159.8)	39	0.95 (−0.27; 2.17)	0.13	13	−0.12 (−1.33; 1.10)	0.85	–	–	–
	p for trend		*0.13*			*0.67*				

β (95% CI): regression coefficient (95% confidential interval). [a]adjusted for maternal age (year), pre-pregnancy BMI (kg/m²), educational level, alcohol intake during pregnancy and parity; [b]adjusted for maternal age (year), pre-pregnancy BMI

(kg/m²), education level, alcohol intake during pregnancy, parity and gestational age at birth. Bold indicates statistical significance ($p \leq 0.050$), # borderline significance ($p \leq 0.080$).

Table S7. The association of serum AhR-TEQ (pg/g lipid) and foetal growth indices stratified by gestation age in ACCEPT cohort.

		Crude			Adjusted[a]		
	**AhR-TEQ (pg/g lipid)**	n	β (95% CI)	*p*	n	β (95% CI)	*p*
**Gestational age ≥ 37 weeks**							
Birth weight (g)	Continuous	408	−0.42 (−0.81; −0.03)	**0.04**	230	−0.25 (−0.77; 0.28)	0.35
	Q1 (0.224–34.20)	94	0 (reference)		56	0 (reference)	
	Q2 (34.21–86.20)	103	76.1 (−63.1; 215)	0.28	66	89.0 (−81.7; 260)	0.31
	Q3 (86.21–159.8)	106	71.4 (−55.0; 198)	0.27	61	133 (−43.5; 310)	0.14
	Q4 (> 159.8)	105	−40.1 (−175; 94.7)	0.56	47	−53.3 (−259; 152)	0.61
	p for trend		*0.53*			*0.88*	
Birth length (cm)	Continuous	408	−0.002 (−0.003; 2e⁻⁴)	0.07#	230	−0.001 (−0.004; 0.001)	0.24
	Q1 (0.224–34.20)	94	0 (reference)		56	0 (reference)	
	Q2 (34.21–86.20)	103	0.41 (−0.19; 1.01)	0.18	66	0.30 (−0.44; 1.03)	0.43
	Q3 (86.21–159.8)	106	0.38 (−0.21; 0.98)	0.21	61	0.26 (−0.54; 1.06)	0.53
	Q4 (> 159.8)	105	−0.24 (−0.84; 0.36)	0.44	47	−0.40 (−1.32; 0.51)	0.39
	p for trend		*0.41*			*0.36*	
Ponderal Index (g/cm³)	Continuous	408	$-8.1e^{-5}$ ($-3e^{-4}$; $2e^{-4}$)	0.41	230	$-1.9e^{-5}$ ($-3e^{-4}$; $2e^{-4}$)	0.88
	Q1 (0.224–34.20)	94	0 (reference)		56	0 (reference)	
	Q2 (34.21–86.20)	103	−0.02 (−0.08; 0.05)	0.60	66	0.01 (−0.07; 0.10)	0.75
	Q3 (86.21–159.8)	106	−0.006 (−0.08; 0.06)	0.86	61	0.06 (−0.04; 0.15)	0.24
	Q4 (> 159.8)	105	$7.8e^{-5}$ (−0.07; 0.07)	0.99	47	0.01 (−0.09; 0.05)	0.83
	p for trend		*0.90*			*0.45*	
Head circumference (cm)	Continuous	407	−0.001 (−0.002; $2e^{-4}$)	0.13	230	−0.001 (−0.002; 0.001)	0.42
	Q1 (0.224–34.20)	94	0 (reference)		56	0 (reference)	
	Q2 (34.21–86.20)	102	0.26 (−0.15; 0.68)	0.21	66	0.42 (−0.11; 0.95)	0.12
	Q3 (86.21–159.8)	106	0.29 (−0.07; 0.65)	0.11	61	0.30 (−0.18; 0.79)	0.22
	Q4 (> 159.8)	105	−0.06 (−0.44; 0.32)	0.74	47	−0.04 (−0.59; 0.51)	0.88
	p for trend		*0.74*			*0.68*	
Gestational age at birth (week)	Continuous	408	−0.001 (−0.002; $3e^{-4}$)	0.16	230	−0.001 (−0.002; $4e^{-4}$)	0.15
	Q1 (0.224–34.20)	94	0 (reference)		56	0 (reference)	
	Q2 (34.21–86.20)	103	−0.13 (−0.48; 0.22)	0.48	66	0.01 (−0.44; 0.46)	0.97
	Q3 (86.21–159.8)	106	0.16 (−0.19; 0.50)	0.37	61	0.10 (−0.36; 0.57)	0.66
	Q4 (> 159.8)	105	−0.25 (−0.60; 0.10)	0.16	47	−0.34 (−0.81; 0.13)	0.16
	p for trend		*0.43*			*0.23*	
**Gestational age < 37 weeks**							
Birth weight (g)	Continuous	24	−0.46 (−3.27; 2.36)	0.74	16	−2.55 (-6.75; 1.66)	0.20
Birth length (cm)	Continuous	24	−0.002 (−0.03; 0.02)	0.88	16	−0.03 (-0.07; 0.01)	0.12
Ponderal Index (g/m³)	Continuous	24	$4.2e^{-5}$ (−0.003; 0.003)	0.97	16	0.003 (−0.001; 0.008)	0.15
Head circumference (cm)	Continuous	24	0.001 (−0.01; 0.01)	0.88	16	−0.007 (−0.02; 0.01)	0.38
Gestational week at birth	Continuous	24	0.002 (−0.01; 0.02)	0.76	16	−0.009 (−0.03; 0.01)	0.32

β (95% CI): regression coefficient (95% confidential interval). [a]adjusted for maternal age (year), pre-pregnancy BMI (kg/m²), plasma cotinine, alcohol intake during pregnancy, education level and parity. Bold value indicates statistical significance ($p \leq 0.050$), # borderline significance ($p \leq 0.080$).
